# Integrative analyses on the ciliates *Colpoda* illuminate the life history evolution of soil microorganisms

**DOI:** 10.1128/msystems.01379-23

**Published:** 2024-05-31

**Authors:** Haichao Li, Kun Wu, Yuan Feng, Chao Gao, Yaohai Wang, Yuanyuan Zhang, Jiao Pan, Xiaopeng Shen, Rebecca A. Zufall, Yu Zhang, Weipeng Zhang, Jin Sun, Zhiqiang Ye, Weiyi Li, Michael Lynch, Hongan Long

**Affiliations:** 1 Key Laboratory of Evolution and Marine Biodiversity (Ministry of Education), Institute of Evolution and Marine Biodiversity, Ocean University of China, Qingdao, Shandong Province, China; 2 Laboratory for Marine Biology and Biotechnology, Qingdao Marine Science and Technology Center, Qingdao, Shandong Province, China; 3 College of Life Sciences, Anhui Normal University, Wuhu, Anhui Province, China; 4 Department of Biology and Biochemistry, University of Houston, Houston, Texas, USA; 5 School of Mathematics Science, Ocean University of China, Qingdao, Shandong Province, China; 6 School of Life Sciences, Central China Normal University, Wuhan, Hubei Province, China; 7 Department of Genetics, Stanford University School of Medicine, Stanford, California, USA; 8 Biodesign Center for Mechanisms of Evolution, Arizona State University, Tempe, Arizona, USA; University of Massachusetts Amherst, Amherst, Massachusetts, USA

**Keywords:** life history, microbial evolution, protists, population genomics, functional genomics

## Abstract

Microorganisms play a central role in sustaining soil ecosystems and agriculture, and these functions are usually associated with their complex life history. Yet, the regulation and evolution of life history have remained enigmatic and poorly understood, especially in protozoa, the third most abundant group of organisms in the soil. Here, we explore the life history of a cosmopolitan species—*Colpoda steinii*. Our analysis has yielded a high-quality macronuclear genome for *C. steinii*, with size of 155 Mbp and 37,123 protein-coding genes, as well as mean intron length of ~93 bp, longer than most other studied ciliates. Notably, we identify two possible whole-genome duplication events in *C. steinii*, which may account for its genome being about twice the size of *C. inflata’s*, another co-existing species. We further resolve the gene expression profiles in diverse life stages of *C. steinii*, which are also corroborated in *C. inflata*. During the resting cyst stage, genes associated with cell death and vacuole formation are upregulated, and translation-related genes are downregulated. While the translation-related genes are upregulated during the excystment of resting cysts. Reproductive cysts exhibit a significant reduction in cell adhesion. We also demonstrate that most genes expressed in specific life stages are under strong purifying selection. This study offers a deeper understanding of the life history evolution that underpins the extraordinary success and ecological functions of microorganisms in soil ecosystems.

**IMPORTANCE:**

*Colpoda* species, as a prominent group among the most widely distributed and abundant soil microorganisms, play a crucial role in sustaining soil ecosystems and promoting plant growth. This investigation reveals their exceptional macronuclear genomic features, including significantly large genome size, long introns, and numerous gene duplications. The gene expression profiles and the specific biological functions associated with the transitions between various life stages are also elucidated. The vast majority of genes linked to life stage transitions are subject to strong purifying selection, as inferred from multiple natural strains newly isolated and deeply sequenced. This substantiates the enduring and conservative nature of *Colpoda*’s life history, which has persisted throughout the extensive evolutionary history of these highly successful protozoa in soil. These findings shed light on the evolutionary dynamics of microbial eukaryotes in the ever-fluctuating soil environments. This integrative research represents a significant advancement in understanding the life histories of these understudied single-celled eukaryotes.

## INTRODUCTION

The life history of an organism refers to its survival and reproductive patterns, including traits that directly impact survival and the timing or quantity of reproduction (1). The optimization of trade-offs linked to growth, reproduction, and survival through natural selection has shaped life history traits. Consequently, organisms sharing the same phylogeny tend to have similar traits (2). The life history strategies of soil microorganisms are highly diverse, endowing them with the exceptional capacity to thrive in a variety of environments, such as terrestrial soils, freshwater, and oceans. However, despite these, the associated regulatory mechanisms and evolutionary processes have received little research attention. *Colpoda* organismsare ciliates prevalent in diverse habitats worldwide, with a particularly notable presence in soil ecosystems. *Colpoda* species share a common feature typical of all ciliates—the presence of a dimorphic nuclear apparatus, comprising both micronucleus and macronucleus sequestered in the same cytoplasm. To date, a total of 32 nominal species of *Colpoda* have been documented, and their cosmopolitan distribution has been empirically substantiated through an extensive body of global investigations (3–6). Moreover, they show highly similar life histories, characterized by distinct life stages, including trophonts—cells in a state of vegetative growth when food bacteria are abundant, reproductive cysts—a mode of asexual reproduction within a cyst wall that distinguishes them from most other ciliates (7), and resting cysts—a dormant stage that helps endure harsh and highly fluctuating soil environments (7, 8). Importantly, these resting cysts are transformable with trophonts. The encystment process is widely acknowledged as a key mechanism contributing to the near-global distribution of *Colpoda* (8).

*Colpoda* species are well-known for their ease of collection and significant agricultural contributions, such as promoting the growth of crops like corn (9), cucumber (10), and rice (11). They also serve as valuable indicator species for monitoring soil quality and environmental pollution (12). The morphological features of *Colpoda* have been extensively documented in previous studies (13–15). For example, the transformation of trophonts into resting cysts includes oral absorption, shedding of cilia, filling of small vesicles within the cyst, and the formation of the resisting cyst wall (16, 17). Despite resting cysts are crucial in the life history of *Colpoda*, there are only a few studies focusing on their molecular mechanisms. For example, Sogame et al. (18, 19) proposed the involvement of elongation factor 1 (EF-1α) in inhibiting resting cyst formation. Furthermore, an increase in cAMP concentration, achieved through adenylate cyclase activation, has been shown to promote encystment (20). Additionally, Jiang et al. (21) applied transcriptomic analyses to investigate gene expression involved in the formation of resting cysts. Their findings revealed a reduction in biosynthesis and energy metabolism, coupled with a significant upregulation of autophagy during this critical stage. A systematic exploration encompassing various life stage transitions using omics tools remains an area yet to be investigated.

**Fig 1:**
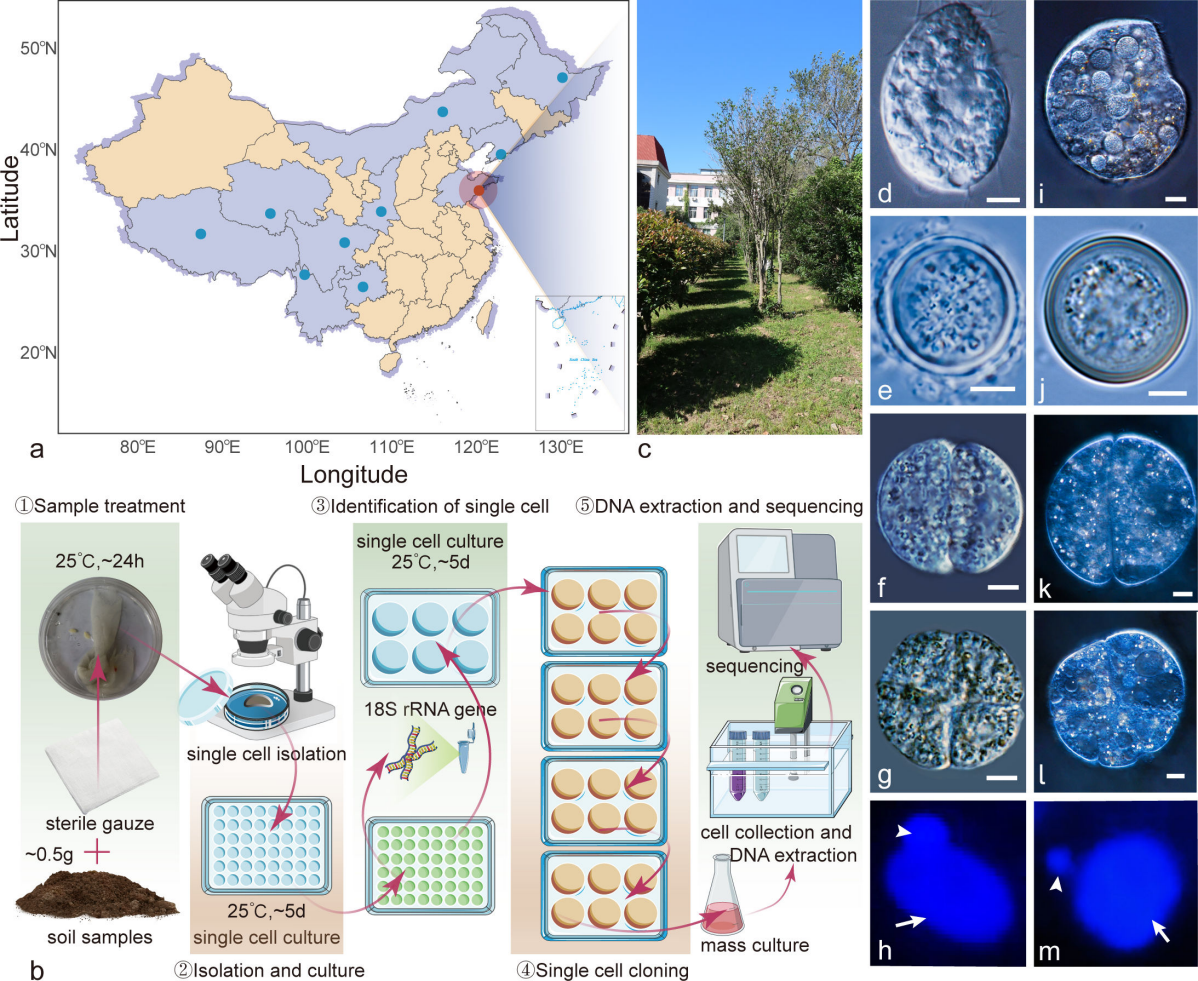
Life history, sample collection and preparation, and morphology of *Colpoda* spp. (**a**) Sampling sites of the 10 natural strains of *C. steinii*. Blue dots represent nine isolates used for population genomic analysis, and the orange dot shows the reference strain for the *de novo* assembly. (Map from https://datav.aliyun.com/portal/school/atlas/area_selector#&lat=30.332329214580188&lng=106.72278672066881&zoom=3.5.) (**b**) Strain isolation and preparation for genomics. (**c**) The sampling site of *C. steinii* and *C. inflata*. (d–g) Photomicrographs of *C. steinii* RZ4A at different life stages: d, trophonts in vegetative growth; e, resting cysts; f, g, reproductive cysts with two and four cells. (i–l) Photomicrographs of *C. inflata* RL4B at different life stages: i, trophonts; j, resting cysts; k, l, reproductive cysts. Scale bar represents 5 µm. (**h and m**) DAPI staining of the nuclei of *Colpoda*: h, *C. steinii* RZ4A; m, *C. inflata* RL4B. Arrow shows the macronucleus, and arrowhead marks the micronucleus.

Cell divisions through the idiosyncratic formation of reproductive cysts in *Colpoda* (Fig. 1; Movie S1) have received limited attention, primarily investigated at the morphological level, dating back more than half a century (22). The molecular regulation of these cell divisions, albeit underexplored, may offer a valuable context for investigating non-canonical eukaryotic cell cycles involving amitosis and polyploidy (as the macronuclear chromosomes lack centromeres and are polyploid) and cellular differentiation. Similarly, while there are numerous studies on multiple whole-genome duplications, sexual processes involving micronucleus-macronucleus development and mating types, genomics, etc., in ciliates (23–26), corresponding investigations on *Colpoda* are mostly absent. It is worth noting that most of the previous efforts on *Colpoda* were conducted in the last century, and the current number of active researchers dedicated to studying *Colpoda* may be even fewer than the known *Colpoda* species.

Despite the rapid advancement of multi-omics technology, investigations into the life histories of ciliates have primarily focused on a handful of species. These studies have often relied on comparative transcriptomics but typically lacked high-quality *de novo* macronuclear genome assemblies. Such genome assemblies are essential for understanding the genetic basis and enhancing the precision and quantification of differential gene expression analyses, akin to reference-based transcriptome analyses (27–30). *Colpoda* organisms, despite their near-global distribution and vital ecological roles, have remained among the understudied majority, in stark contrast to the genomic resources of model ciliates like *Tetrahymena* and *Paramecium* (23, 31–34).

In this study, we explored the life history of *Colpoda steinii* at both phenotypic and molecular levels. Our approach involved uncovering its molecular basis through *de novo* macronuclear assembly and gene annotation, using Nanopore long-read sequencing. We also revealed differential gene expression by performing low-input RNA library preparation across various life stages, conducted comparative genomics with the *de novo* assembled macronuclear genomes of another co-existing congener *C. inflata*, as well as published genomes of distantly-related ciliates, investigated population genomics using strains newly collected from diverse regions in China, and examined the proteomics of resting cysts. This study will provide new data to uncover the strategies and evolution of soil ciliates in resisting adverse environments. It also lays the necessary foundation for a deeper understanding of the role of protozoa in maintaining the functioning and long-term evolution of soil ecosystems.

## RESULTS

### *De novo* assembly and gene annotation of the macronuclear genome of *Colpoda steinii* RZ4A

We collected soil samples, isolated single *Colpoda* cells, and identified species by live microscopy and 18S rDNA Sanger sequencing (Fig. 1a through c). To ensure the purification of the cell lines, we executed five rounds of single-cell passaging (Fig. 1b). As a result, we successfully established a clonal cell line, denoted as *C. steinii* RZ4A, which has a typical life history akin to most previously reported *Colpoda* species.

Subsequently, we performed genome sequencing on this strain using Illumina Novaseq 6000 and Oxford Nanopore MinION platforms. Following the exclusion of reads stemming from food bacteria contamination and the removal of low-quality bases or adaptors, we generated a total of 23.89 Gbp Nanopore and 31.54 Gbp Illumina PE150 high-quality sequences (Table 1). We initiated the assembly process by first compiling the Nanopore clean reads into contigs, subsequently merging them into scaffolds. The draft genome was then polished with high-quality Illumina short reads. Based on the distribution of GC content within the scaffolds, we further filtered out scaffolds that potentially originated from bacteria, as well as contaminating scaffolds of micronucleus sources according to sequencing depth. To prevent the inadvertent exclusion of genuine genome components, we conducted a thorough examination of scaffolds that were flagged for removal (see details in Supplemental Materials and Methods).

**TABLE 1:** Macronuclear genomic features of *C. steinii* RZ4A and *C. inflata* RL4B

Features	*C. steinii* RZ4A	*C. inflata* RL4B
Illumina PE150 sequences (Gbp)	31.54	34.67
Nanopore sequences (Gbp)	23.89	19.23
Genome size (bp)	155,392,961	81,751,086
Number of scaffolds	7,989	2,481
Number of scaffolds (≥1,000 bp)	7,961	2,480
Number of scaffolds (≥10,000 bp)	3,738	2,041
Number of scaffolds (≥50,000 bp)	710	385
Largest scaffolds	501,730	1,462,395
N50 (bp)	40,553	46,401
GC content (%)	33.55	35.09
Telomere	TT(T/G)GGG	TT(T/G)GGG
Scaffolds with telomere (%)	82.05	58.52
2-Telomeres scaffolds	2,717	307
1-Telomere scaffolds	3,838	1,145
BUSCO (%)	90.1	76.6
Number of genes	37,123	22,668
Mean gene size (bp)	2,034	1,876
CDS size (bp)	440	456
Number of introns	110,669	59,429
Genes with introns	22,652	12,430
Mean intron size (bp)	93.2	85.01
Mean no. of introns	2.98	2.62

Ultimately, our efforts yielded a high-quality macronuclear genome for *C. steinii* RZ4A, with 155.39 Mbp in length, N50 of 40,553 bp (most ciliates are known to have short chromosomes in the macronucleus due to chromosome fragmentation), a genome completeness of 90.05% according to the BUSCO assessment (Fig. S1). The genome assembly comprises 7,989 scaffolds, of which 2,717 (~34% of all scaffolds) possess 2 telomeres, while 3,838 (~48%) have 1 telomere (Table 1).

We conducted gene structural annotation through a combination of *de novo* gene prediction and RNAseq evidence. To capture the transcriptome encompassing as many genes as possible, we performed low-input RNAseq on three distinct life stages: trophonts, reproductive cysts, and resting cysts (Fig. 1d through g). This effort resulted in the annotation of 37,123 protein-coding genes, with a mean size of 2,034 bp, featuring 2.98 introns (mean intron size ~93 bp, much longer than those in other ciliates) and 3.98 exons per gene (Table 1).

In terms of gene functional annotation, an exceptional 97.06% of gene models show homologous matches against the NCBI NR database. In addition, 63.03% of genes can be successfully annotated in Gene Ontology (GO). These results collectively demonstrate the high quality of the functional annotation of the genome.

### Transcription and translation profiles associated with the life history

To uncover the transcriptional profiles associated with the life stages of *C. steinii* RZ4A, we first isolated ~20 cells from each distinct life stage on ice with a high-power Olympus dissection microscope. Following isolation, we promptly proceeded with low-input RNA library construction. We conducted an intensive analysis of differential gene expressions across various life stages, using the low-input RNAseq data obtained from *C. steinii* RZ4A, including trophonts in vegetative growth, reproductive cysts, resting cysts, and revived trophonts, with each stage comprising a minimum of at least three replicates. To ensure the reliability of our RNAseq data sets, we performed rigorous quality control assessments by principal component and clustering analyses. The identification of significantly differentially expressed genes (DEGs) between two life stages was executed using DESeq2, with a defined threshold of |fold-change| > 2 and *P*_adj_ < 0.05.

#### Resting cysts vs trophonts

**Fig 2:**
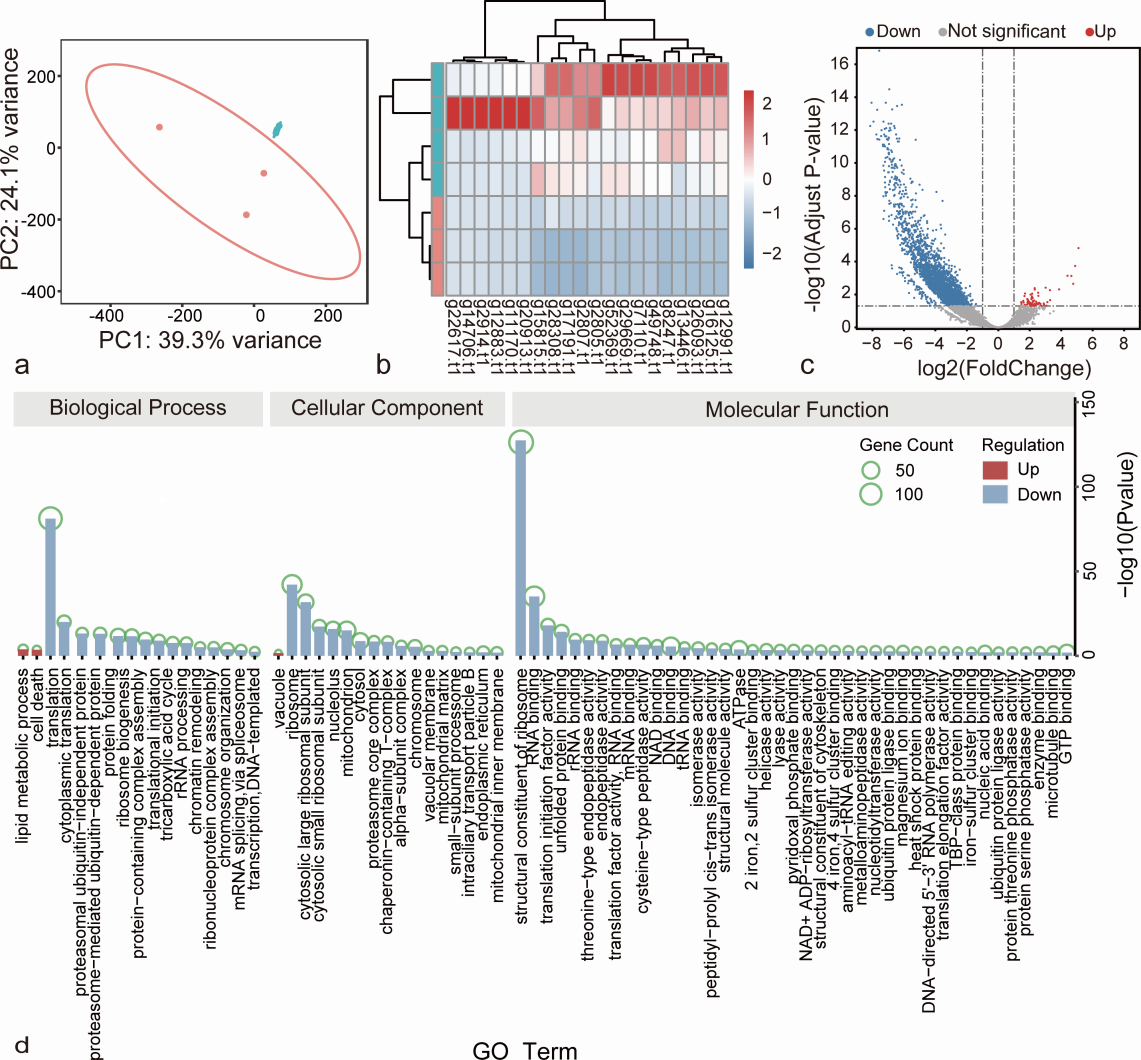
Differential gene expression and gene enrichment analyses of resting cysts vs trophonts in *C. steinii* RZ4A. (**a**) Principal component analysis of mRNAs of trophonts and resting cysts. Each dot represents one sample. Blue dots are resting cyst samples, and red ones are trophont samples. (**b**) The heatmap of top 20 differentially expressed genes in resting cysts (vs trophonts). The blue rectangles at the far left of the figure are resting cyst, and the red are trophont. (**c**) DEGs in resting cysts (vs trophonts). Gray, red, and blue dots represent not significantly, significantly up- and downregulated DEGs. (**d**) Results from GO enrichment analyses, using up- or downregulated DEGs.

We compared resting cysts with trophonts, with their replicated samples clustered together (Fig. 2a). There were significant distinctions in overall gene expression between the two life stages (Fig. 2b), and we found that 69 genes were upregulated and 2,313 downregulated in resting cysts (Fig. 2c; Table S1). To gain deeper insights into the biological processes, molecular functions, and cell components involved in the life stage transition of *C. steinii* RZ4A, we performed GO enrichment analysis focusing on the DEGs between these life stages.

Consistent with previous observations indicating that resting cysts represent a dormant stage, we detected a significant downregulation in their major cellular activities, particularly genes associated with translation (Fig. 2d). As expected, resting cysts still maintain essential metabolic functions, as evidenced by the slight background expression observed in most genes. This was further corroborated by our proteome sequencing on resting cysts, which identified 1,483 peptide hits corresponding to genes associated with translation, protein folding, microtubule cytoskeleton organization, and other processes (Tables S1 and S2; Fig. S2). Notably, the abundance of LamG-domain-containing proteins was found to be the highest in resting cysts (Table S2). These proteins are usually Ca^2+^-mediated receptors. We hypothesize that these proteins may be the primary receptors for the molecular process of the formation of resting cysts because the regulation of Ca^2+^ concentration can induce the formation of resting cysts in *Colpoda* (20).

*Colpoda* species are also known for their propensity to generate numerous small vesicles and vacuoles within the cytoplasm during the formation of resting cysts, a phenomenon well-documented. In the case of *C. steinii* RZ4A, we have observed that upregulated genes are enriched in lipid metabolism (GO:0006629) and the vacuole cellular component (GO:0005773) (Fig. 2d). According to previous studies, the extrusion of macronuclear chromatin is a distinctive feature in the process of encystment in a manner similar to the apoptosis-like nuclear death that occurs during conjugation in other ciliates (13). In line with this, we have also identified two additional upregulated genes (g20033.t1 and g7555.t1) enriched in cell death (GO:0008219; Fig. S3). Our results align nicely with the aforementioned conjectures suggesting that events similar to cell death may occur during encystment.

These findings collectively reinforce the reliability of our differential gene expression analyses based on RNAseq. Furthermore, they provide valuable molecular insights into the process of encystment—a critical survival strategy used by *Colpoda* to contend with the challenging and ever-changing conditions of their soil habitats.

#### Revived trophonts vs resting cysts

When an ample supply of food bacteria becomes available, resting cysts undergo excystment and transition into trophonts. In our experimental setup, we initiated this process by re-introducing food bacteria to the resting cysts of *C. steinii* RZ4A. Remarkably, within a span of 12 h following this re-introduction, the resting cysts commenced excystment, giving rise to trophonts. The resting cysts before re-feeding and newly formed trophonts were promptly isolated and used for the construction of low-input RNAseq libraries, which were subsequently subject to Illumina PE150 sequencing.

**Fig 3:**
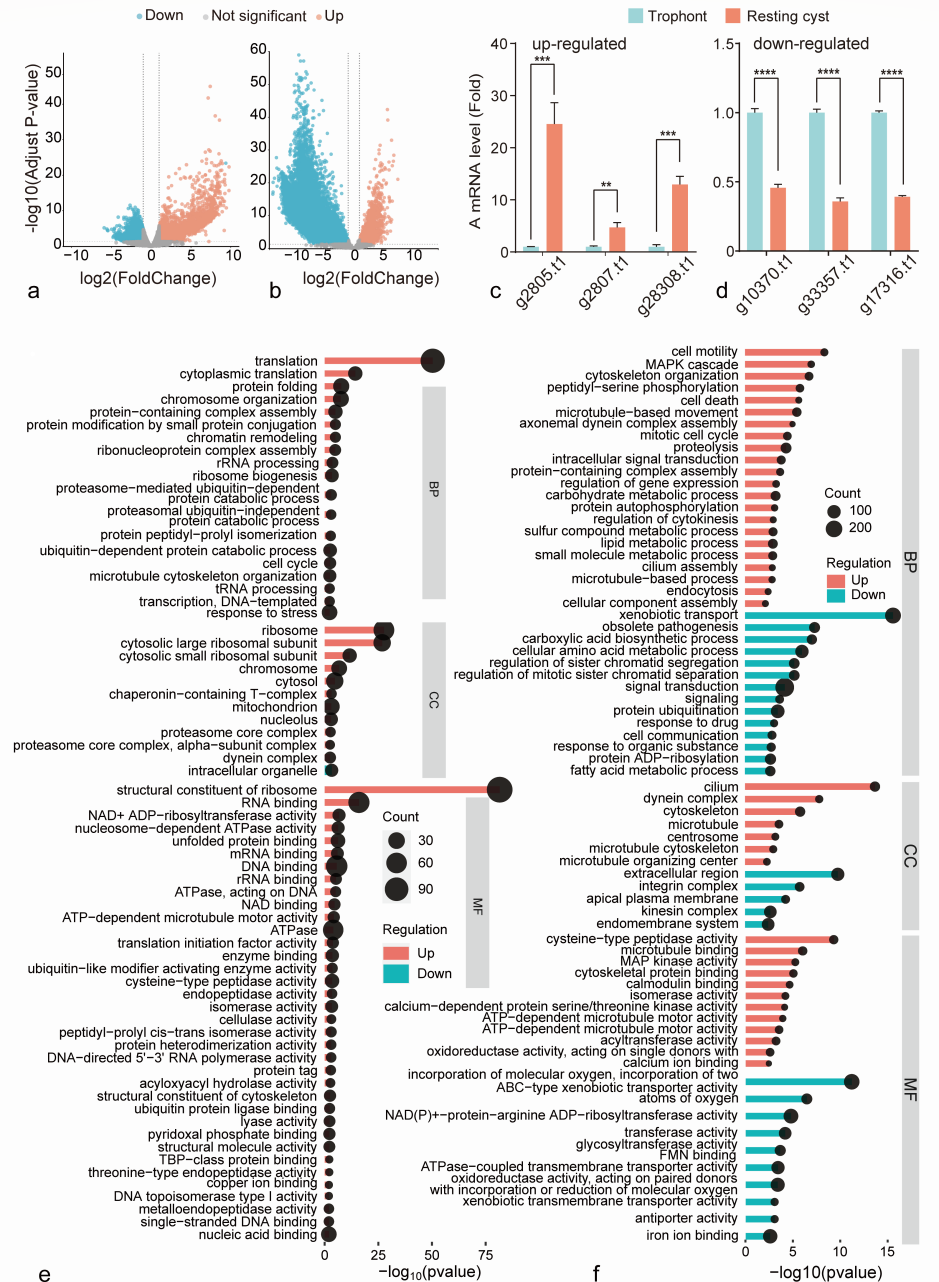
Differential gene expression of revived trophonts and reproductive cysts in *C. steinii* RZ4A. (**a**) Differential expression profiles of revived trophonts (vs resting cysts) and (**b**) reproductive cysts (vs trophonts in vegetative growth). Gray, red, and blue dots represent not significantly, significantly up- and downregulated DEGs, respectively. (**c and d**) RT-qPCR verification of DEGs in resting cysts (vs trophonts). **, ***, and **** represent *P* value < 0.01, 0.001, and 0.0001. (**e**) GO functional enrichment of DEGs of revived trophonts (vs resting cysts) and (f) reproductive cysts (vs trophonts). The size of the black dots represents the number of enriched genes in each pathway.

Through an intensive analysis of differential gene expression, we identified a total of 2,803 upregulated and 2,486 downregulated genes in the revived trophonts when compared to the resting cysts (Fig. 3a; Table S3). We detected active expression of translation-related genes in the revived trophonts (Fig. 3e), and they are downregulated in the resting cysts (vs trophonts). By comparing the upregulated genes in the revived trophonts with those downregulated in the resting cysts, we found that more than one-third of the genes were shared between them, and these genes may play a crucial role in the mutual transformation between trophonts and resting cysts.

#### Reproductive cysts vs trophonts

Similar to other *Colpoda* congeners, *C. steinii* RZ4A reproduces by forming reproductive cysts, which typically give rise to two or four trophonts upon bursting (Fig. 1f and g; Movie S1). Using differential gene expression analyses, we have identified a total of 2,297 genes that are upregulated in reproductive cysts when compared to trophonts (Fig. 3b; Table S4). These genes are enriched in molecular functions commonly associated with eukaryotic cell division, encompassing processes such as mitosis, protein phosphorylation, MAPK cascade activation, lipid metabolism, and so on. Furthermore, they are associated with critical cell components including cilia, microtubules, spindles, centromeres, and others (Fig. 3f).

Among these molecular functions, those related to cilia are particularly prominent (Fig. 3f), indicating the heightened activity in the generation and movement of cilia of progeny cells enclosed within the reproductive cyst wall. By contrast, there is a noticeable reduction in genes associated with the transport of xenobiotic and communication with the extracellular environment (Fig. 3f). This observation further underscores that the cell-division process within the reproductive cysts is an independent process, reducing the interference of the external environment as much as possible to ensure high-fidelity cell divisions.

#### Technical validation of the DEGs analyses using RT-qPCR

To corroborate the accuracy of our differential gene expression analyses, we first conducted RT-qPCR experiments using newly prepared total RNA of trophonts and resting cysts as templates. Then, we did reverse transcription and qPCR, targeting six randomly chosen genes of *C. steinii* RZ4A (upregulated: g10370.t1, g17316.t1, g33357.t1; downregulated: g2805.t1, g2807.t1, g28308.t1; Table S5). These genes were chosen based on their observed up- or downregulation during the resting cyst stage compared to trophonts, as indicated by our earlier analyses using low-input RNAseq. Encouragingly, the expression patterns of all these genes were found to be consistent with those derived from the low-input RNAseq data sets (Fig. 3c and d). This alignment serves as compelling evidence affirming the reliability of the above differential gene expression analyses.

#### Validation of the gene ontology analyses

In addition, we made efforts to perform RNAi targeting the aforementioned six genes, using the L4440 plasmid system. However, we have not yet achieved success in these endeavors. Fortunately, given the high degree of conservation in the life histories of *Colpoda* species, it is reasonable to anticipate that the molecular mechanisms revealed through our GO analyses in *C. steinii* RZ4A are also likely to be present in congeners.

**Fig 4:**
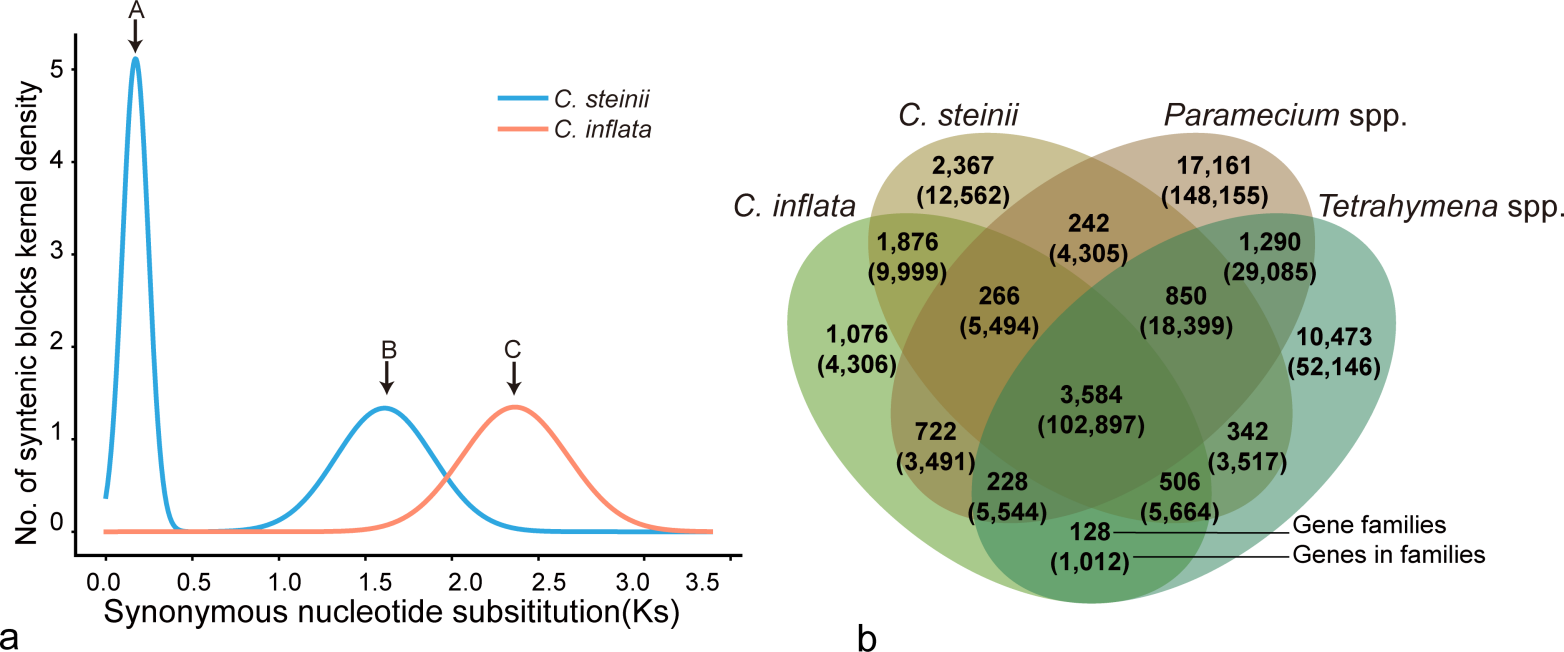
Genome evolution of *Colpoda*. (**a**) The Ks distribution of *C. steinii* RZ4A and *C. inflata* RL4B macronuclear genomes. Blue and orange represent *C. steinii* RZ4A and *C. inflata* RL4B information, respectively. After WGD occurred, paralogs doubled and accumulated mutations in parallel, which increased the synonymous mutations (Ks) and led to Ks peaks. Peaks A and B represent two WGD events in *C. steinii* RZ4A. Peak C refers to the only WGD event in *C. inflata* RL4B. (**b**) Homologous gene cluster analysis. Venn diagram illustrates the shared and unique gene families of *Colpoda*, compared with *Tetrahymena* and *Paramecium*.

We then proceeded to isolate *C. inflata* RL4B from the same sampling site as *C. steinii* RZ4A (Fig. 1c, i through l). Following similar procedures, we first *de novo* assembled and annotated its macronuclear genome. The genome of *C. inflata* RL4B is approximately 81.75 Mbp in size, harboring a total of 22,668 genes, and shares identical telomere sequences [5′-TT (T/G)GGG-3′] with *C. steinii* RZ4A (Table 1). Significantly, there are notable differences in the scaffold count between the macronuclear genomes of the two species, with *C. inflata* RL4B featuring 2,481 scaffolds (with N50 ~46.40 kbp) compared to *C. steinii* RZ4A’s 7,989 scaffolds (with N50 ~40.55 kbp). This discrepancy is consistent with the observation that the genome size of the latter (155.39 Mbp) is roughly twice that of the former (81.75 Mbp) (Table 1). The increase in genome size may be driven by multiple processes, including whole-genome duplication (WGD), transposable elements (TE) proliferation, intron amplification, and tandem gene duplication (35). Using a synteny analysis of homologous gene pairs, we have discerned that two WGD events might have occurred in *C. steinii* RZ4A, whereas only one ancient WGD event in *C. inflata* RL4B (Fig. 4a). This fits nicely with the approximately twofold difference in genome size between the two species.

Using the genome of *C. inflata* RL4B as a reference and harnessing low-input RNAseq data from various life stages, we identified DEGs and conducted GO enrichment analyses. Impressively, numerous DEGs in *C. inflata* RL4B overlapped with those identified in *C. steinii* RZ4A and were enriched in the same molecular functions, biological processes, or cell components as those of *C. steinii* RZ4A. These included the upregulation of genes associated with cell death and vacuole formation, as well as the downregulation of translation in resting cysts (compared to trophonts in vegetative growth) (Table S6). Additionally, we observed significant enrichment of translation-related genes in revived trophonts (in contrast to resting cysts) (Table S7). Moreover, active biological processes, such as microtubule-based movement, cilium assembly, and cell motility, along with a noteworthy downregulation of cell adhesion regulated by the integrin complex, were evident in reproductive cysts (vs trophonts) (Table S8). These findings supported the reliability of our DEGs and GO analyses, as well as the robustness of gene expression patterns during the transformation between life stages.

### Molecular evolution of DEGs associated with different life stages

To elucidate the evolutionary patterns underlying the above DEGs in the transcriptional profiles of different life stages, we first performed gene family expansion and contraction analyses. Since gene family expansion can facilitate adaptation, the expansion of gene families containing DEGs may serve as an indicator of complex life history conferring selective advantages for *Colpoda*. Using 14 previously published high-quality ciliate macronuclear genomes (including 2 *Colpoda* genomes from this study, as well as genomes from 4 *Tetrahymena* and 8 *Paramecium* species; Table S9), a total of 406,576 genes were clustered into 41,111 gene families. Of these, 102,897 genes were allocated into 3,584 shared gene families found in all species, while 1,876 gene families were exclusive to *Colpoda* (Fig. 4b). Our analyses also revealed a total of 19,711 gene duplication events in *C. steinii* RZ4A and 7,587 in *C. inflata* RL4B (Fig. S4), a finding that aligns consistently with the aforementioned WGD analyses.

Among the DEGs identified in resting cysts (in comparison to trophonts in vegetative growth) and in revived trophonts (vs resting cysts) of *C. steinii* RZ4A, 28.72% and 29.32% of them are in expanding gene families, while only 6.05% and 3.37% are in contracting ones (Tables S10 and S11). Significantly, many of the DEGs implicated in encystment and excystment processes are found within these expanding gene families, implying the possibility of these genes conferring selective advantages.

Similarly, for the DEGs identified in reproductive cysts (vs trophonts) of *C. steinii* RZ4A, a substantial 26.67% are associated with expanding gene families, with only 0.99% in contracting ones. It is noteworthy that the majority of these DEGs are involved in the cell adhesion processes (Table S12), suggesting that this molecular function has also been preserved by expanding gene families during the course of evolution.

The gene expansion and contraction analyses presented above provide evidence of DEGs that are subject to selection pressures. To further quantify the selective strength acting on the life-history-associated DEGs in *C. steinii*, we conducted Illumina-PE150 whole-genome sequencing on nine natural strains of *C. steinii* isolated from soils collected in China between 2020 and 2022 (Fig. 1a; Table S13). The clean Illumina reads were aligned to the genome of *C. steinii* RZ4A, and SNPs were called. In total, we identified 1,157,466 SNPs for *C. steinii*. These SNPs yielded nucleotide diversity at all genomic sites (π), nucleotide diversity at silent sites (π_s_), and Tajima’s *D* values of 0.04, 0.06, and 0.18, respectively.

**Fig 5:**
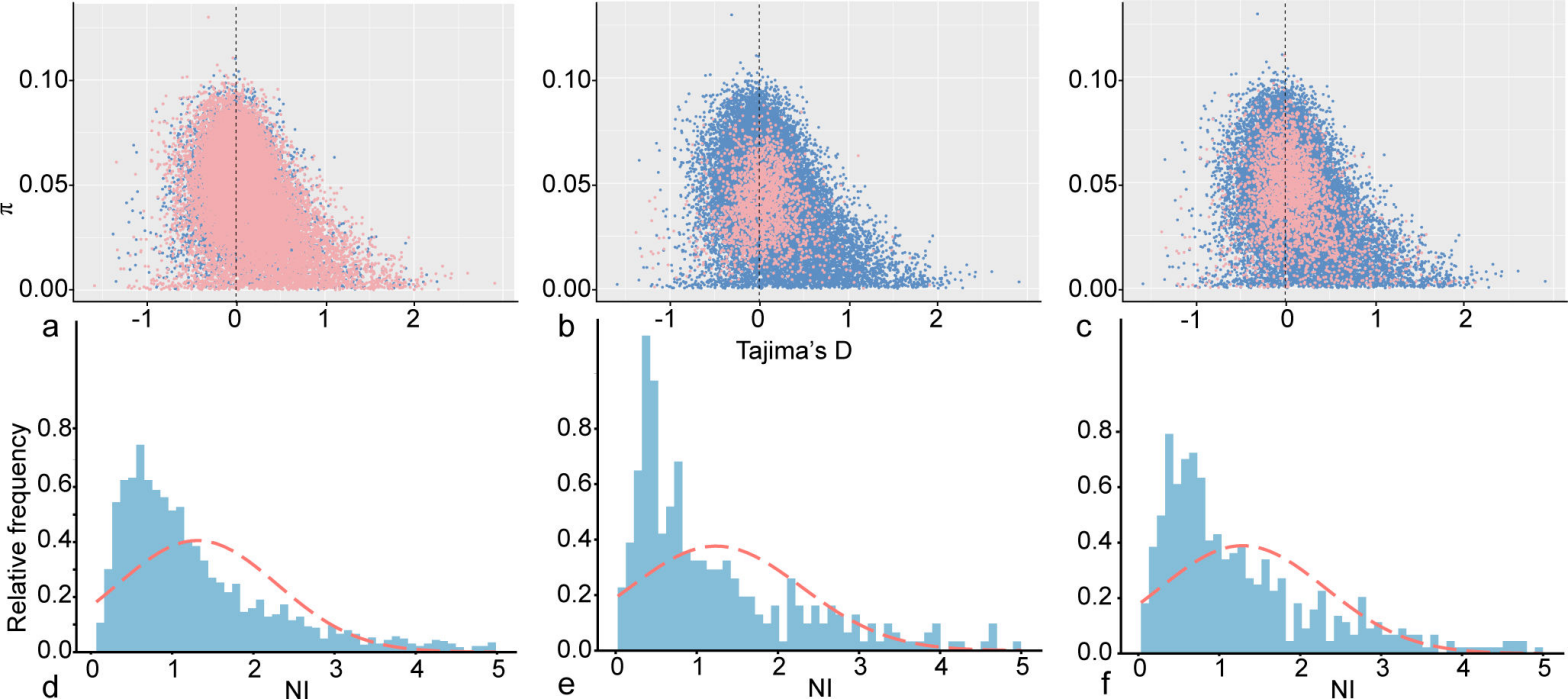
Selective pressures on DEGs of different life stages in *C. steinii* RZ4A. (**a, b, c**) π vs Tajima′s *D* distribution of DEGs in reproductive cysts (vs trophonts, **a**), resting cysts (vs trophonts, **b**), and revived trophonts (vs resting cysts, **c**) of *C. steinii* RZ4A. The blue dots are all the genes in the genome, and the pink dots are DEGs (pink dots show up when they overlap with the blue ones). (**d, e, f**) The distribution of DEGs’ NI values in reproductive cysts (**d**), resting cysts (**e**), and revived trophonts (**f**) of *C. steinii* RZ4A.

Tajima’s *D* test was used to distinguish between random neutral mutations and non-neutral mutations under selective pressure. A Tajima’s *D* value greater than 0 suggests a low frequency of rare alleles, which may be indicative of equilibrium selection pressure or a population undergoing shrinkage/bottleneck. Because mRNA expression levels of the genes specifically expressed during one life stage, regardless of whether they are downregulated or upregulated, do not necessarily correlate with the regulation of associated biological functions. For instance, inhibitors’ expression can be elevated but may result in downstream functional downregulation. We, thus, compiled the DEGs specifically expressed in each life stage as “regulation” DEGs and performed Tajima’s *D* tests on them. Our analyses revealed that the mean Tajima’s *D* for DEGs in reproductive cysts, resting cysts, and revived trophonts are 0.15, 0.03, and 0.09, respectively (Fig. 5a through c). We also used the McDonald-Kreitman (MK) test (a simple and widely used selection test), which is usually done by calculating the mean cross-gene neutrality index (NI) values, to summarize the selection patterns within *C. steinii* (36, 37). The majority of the NI values for the DEGs in reproductive cysts, resting cysts, and revived trophonts are larger than 1, and their distributions are highly similar (Fig. 5d through f). Taken the above together, purifying selection is, thus, dominant in genes associated with life history regulation. This further demonstrates the functional constraint on the complex life history with multiple life stages, as these stages are crucial for survival and reproduction in the highly challenging soil environments.

### Possible sexual processes in *Colpoda*

The presence of sexual processes in *Colpoda* remains a subject of ongoing investigation, as there have been no direct and confirmed reports of conjugative nuclear changes in *Colpoda* to date, a fact also reflected in our own observations since 2018. In a study conducted by Dunthorn et al. (38), they explored 51 meiosis genes (11 meiosis-specific and 40 meiosis-related genes) in *Colpoda magna* and found that most of these genes align with those found in other ciliates known to undergo sexual processes.

**Fig 6:**
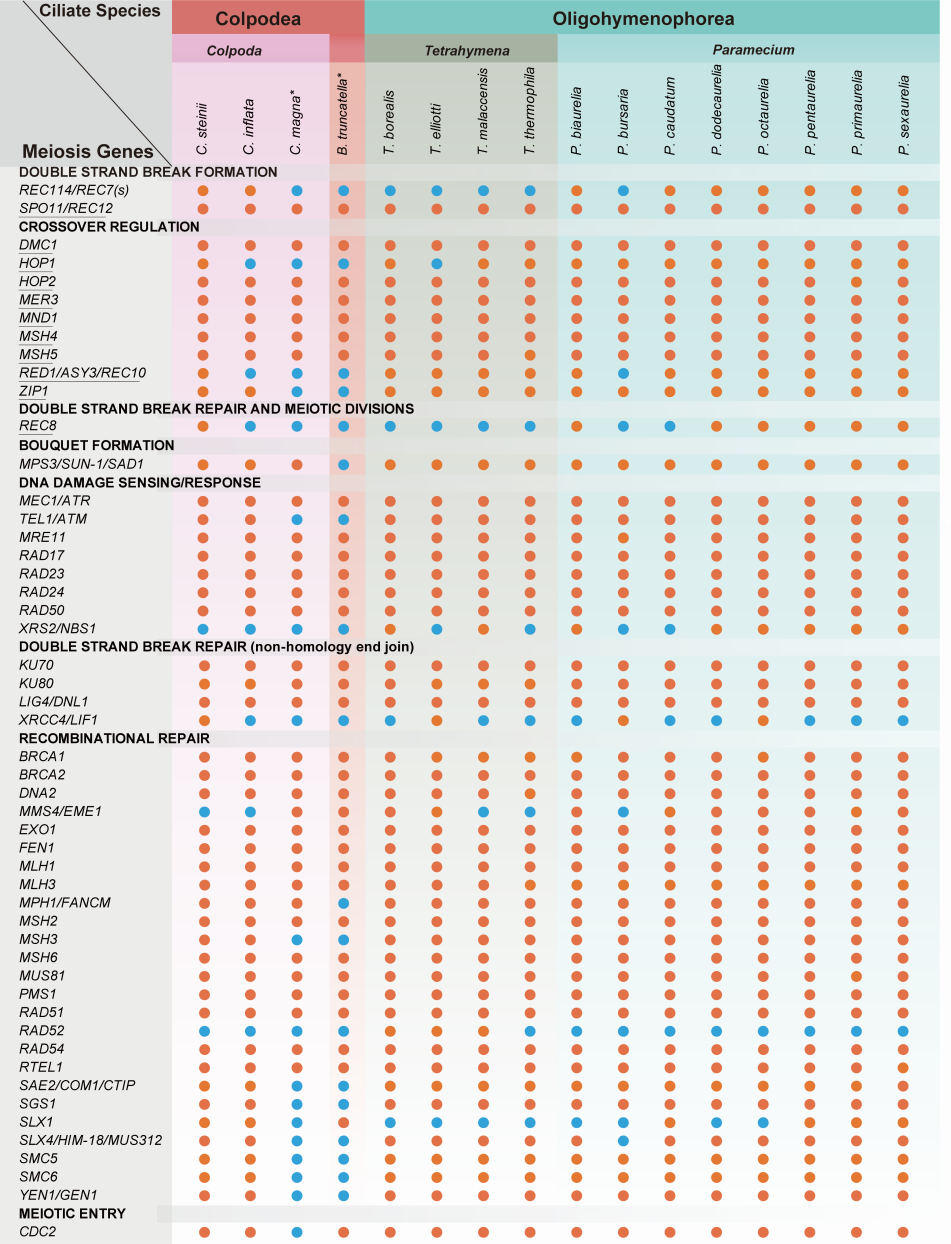
Meiosis genes in 16 ciliate genomes. The red and blue dots represent the presence and absence of genes. The genes underlined are meiosis-specific genes. Species with “*” represent data from Dunthorn et al. (38).

In order to explore this, we followed the study of Dunthorn et al. (38) and reconstructed the database of these 51 meiosis genes, with a total of 391 homologs (Table S14). Subsequently, we conducted a thorough search for these meiosis genes across 16 ciliate macronuclear genomes, including the two *Colpoda* genomes assembled in this study. Our investigations revealed that a substantial portion of meiosis genes are present with intact open reading frames in the macronuclear genome of *C. steinii* RZ4A, including all the 11 meiosis-specific genes that are also commonly found in most *Paramecium* species (indicated by underlined genes in Fig. 6). Among these genes are critical components such as *SPO11*, *DMC1*, *HOP2*, *MER3*, *MND1*, *MSH4,* and *MSH5*, all of which play pivotal roles in recombination processes in eukaryotes (39–42).

Recombination can reduce linkage equilibrium (LD). In order to investigate potential recombination events, we calculated linkage disequilibrium, measured as the squared correlation between allelic states (*r*^2^). Our results revealed a rapid decline of LD within 1 Kbp region, and declining slowly with longer distance (Fig. S5), indicating possible recombination in *C. steinii*. We further evaluated the heterozygosity of the nine *C. steinii* natural strains by calculating method-of-moments *F* coefficient estimates, and found that their *F* values were between 0.56 and 0.87, indicating that homozygosity was more likely. This again raises the possibility of inbreeding or low mutation rate in *Colpoda*. Based on these findings, our results support the notion that all *Colpoda* congeners might undergo cryptic sexual processes, which may rarely occur or not manifest in the form of conjugation. Instead, it is possible that the cryptic sexual process is triggered by as-yet-unknown factors within the natural soil environment.

## DISCUSSION

In this thorough study, our investigation spanned multiple domains of technology, including genomics (encompassing *de novo* macronuclear genome assembly and annotation—first effort using long-read sequencing for the Colpodea class), proteomics, and transcriptomics across four distinct life stages (namely, resting cysts, trophonts in vegetative growth, reproductive cysts, and revived trophonts from resting cysts) using low-input RNAseq, as well as comparative genomics and population-genomics analyses.

We successfully identified a substantial number of DEGs associated with various life stages and confirmed their occurrence during life history of the co-existing congener *C. inflata*; however, it is imperative to further determine their causal effects. Specifically, identifying the trigger genes responsible for transitioning between life stages remains to be done. Functional validation using efficient RNAi or gene-editing tools is the next logical step in our research trajectory despite our unsuccessful preliminary trials. The resolution of these critical aspects holds the promise of providing deeper insights into the evolutionary mechanisms of cell differentiation in these unicellular eukaryotes.

As one of the most successful eukaryotic genera in soil, *Colpoda* has diverse life history strategies that are conserved across taxa (6). These strategies, including the extreme tolerance of resting cysts to harsh environmental conditions and fission within reproductive cysts, likely contribute significantly to their broad distribution and abundant presence (3). Moreover, research has demonstrated that *Colpoda* species play a pivotal role in enhancing nutrient absorption by plants, while the presence of plants, in turn, increases the density of *Colpoda* (9, 43). This mutualistic relationship fosters both the robust growth of plants and the extensive proliferation of *Colpoda* populations.

In addition, the mean guanine-cytosine (GC) content of *Colpoda* genomes exceeds that of most other ciliates (Fig. S6). The high GC composition contributes to genome stability and resilience in extreme environments. Various mechanisms underpin this stability, including the presence of an extra hydrogen bond in G:C base pairs compared to A:T base pairs, and the fact that codons with higher GC encode more hydrophobic amino acids. These hydrophobic amino acids may enhance the stability and resistance to denaturation of proteins within *Colpoda*, reinforcing their adaptability to challenging conditions (44). Numerous predators and highly variable physicochemical factors such as moisture, pH, and temperature might exert much stronger natural selection pressure on *Colpoda* in soil habitats compared to freshwater environments where *Paramecium* and *Tetrahymena* live. These extra selection pressures might have likely contributed to the evolution of higher GC levels in *Colpoda* genomes (45). While *Colpoda* populations sharing the same ecological niches in soil may have undergone co-evolution, the specific interactions between them, including aspects such as competition and mutualism, remain largely unexplored. Future research in this domain promises to reveal additional strategies within *Colpoda*’s toolkit.

While *C. inflata* RL4B experienced a single whole-genome duplication (WGD) event, *C. steinii* RZ4A, by contrast, underwent two such events. This difference in WGD history likely contributes significantly to the observed doubling in genome size between the two *Colpoda* species. The molecular functions of both the expanded gene families and genes retained after WGD in *C. steinii* RZ4A are associated with ATP-binding, ion-binding, and protein-binding proteins (Fig. S4, S7a and b), which are the basic cell functions to maintain vital activities. We speculate that *Colpoda* require intensive energy supply due to the harsh habitats in soil, which may be related to ATP binding. Ion-binding function may be related to morphological changes during the formation of resting and reproductive cysts in *Colpoda*.

The macronuclear genome size of *C. steinii* RZ4A is larger than that of most other ciliates (Fig. S4). Genome size evolution is a multifaceted process influenced by factors such as natural selection, genetic drift, and mutations. These factors collectively contribute to significant changes in genome size (46). In the case of *C. steinii* RZ4A, its genome has experienced the most gene duplications among all the 14 ciliate genomes analyzed in this study, while this could not solely account for its large genome size. For example, in *Paramecium*, there are large genomes with few gene duplications or small genomes with numerous gene duplications (Fig. S4). These findings underscore the intricate nature of genome size evolution, wherein gene duplications can, indeed, lead to genome size expansion, but the roles of natural selection and genetic drift should not be overlooked. Further investigations are needed to disentangle the complex interplay of these genetic factors in shaping genome sizes.

Microbial eukaryotes, comprising a substantial portion of Earth’s organisms, play active roles in the global biogeochemical processes that govern our soils. Their indispensability in the microbial loop, where they facilitate the transfer of materials and energy from bacteria to higher trophic levels, underscores their profound ecological significance. Despite ongoing research efforts, there remains an imperative need for further investigations to unravel the intricate survival strategies embedded in their life histories, alongside the genetic and evolutionary mechanisms that underpin their success. These diminutive yet exceptionally resilient and ancient denizens hold the key to unlocking the mysteries hidden beneath the Earth’s surface at the molecular level, offering fresh insights that extend beyond the limitations of conventional methodologies. In summary, this research represents a significant advancement in the study of the life histories of these understudied single-celled eukaryotes. It does so by using state-of-the-art “-omics” tools, coupled with extensive collections, isolations, microbiological manipulations, and advanced microscopy techniques. This study, thus, paves the way for a more profound understanding of the life history evolution of soil microorganisms.

## MATERIALS AND METHODS

We collected two *Colpoda* species (*C*. *steinii* reference strain RZ4A and the *C. inflata* RL4B) from the Yushan campus of Ocean University of China (36°3′42″N, 120°19′56″E; Fig. 1c), Qingdao, China, on 22 August 2018. Their DNA and RNA were extracted using the MasterPure Complete DNA and RNA Purification kit. Subsequently, we used MinION (in-lab) and NovaSeq6000 (Berry Genomics, Inc., Beijing) sequencers for Nanopore and Illumina sequencing on the reference strains. We then assembled and annotated the macronuclear genomes of both species. In order to reveal transcription patterns of different life stages of each species, low-input RNAseq was performed, followed by differential gene expressions analyses. In addition, we also conducted comparative genomics and population genomics analyses to elucidate the evolutionary patterns of life-history-associated genes. Details can be found in the Supplemental Materials and Methods.

## Data Availability

All FASTQ sequences are available at NCBI BioProject PRJNA937028. The Illumina reads and Nanopore reads of *C. steinii* RZ4A and *C. inflata* RL4B are available with the Biosample IDs of SAMN33386881 and SAMN33386882. The nine NCBI SRA samples (accession numbers SAMN36899794 to SAMN36899802) link FASTQ files containing sequences from natural strains for population analyses. The genome assemblies and annotations of *C. steinii* RZ4A and *C. inflata* RL4B are available at the CNCB (China National Center for Bioinformation) BioProject PRJCA018943 with Biosample IDs of SAMC2987422 and SAMC2989089.

## SUPPLEMENTAL MATERIAL

Supplemental Figures msystems.01379-23-s0001.pdf Figures S1-S8.

Supplemental Text msystems.01379-23-s0002.docx Supplemental Materials and Methods.

Legend msystems.01379-23-s0003.docx Legend for Movie S1.

Supplemental Tables msystems.01379-23-s0004.xlsx Tables S1 and S2.

Additional Supplemental Tables msystems.01379-23-s0005.xlsx Tables S4 to S16.

Table S3 msystems.01379-23-s0006.xlsx Differential gene expression analysis.

Movie S1 msystems.01379-23-s0007.mpeg Division process of the reproductive cysts of *C. steinii*.

ASM does not own the copyrights to Supplemental Material that may be linked to, or accessed through, an article. The authors have granted ASM a non-exclusive, world-wide license to publish the Supplemental Material files. Please contact the corresponding author directly for reuse.
