# Supplementary material for: Integrative analyses on the ciliates Colpoda illuminate the life history evolution of soil microorganisms
Source: mSystems. 2024 May 31;9(6):e01379-23. doi: 10.1128/msystems.01379-23 (PMC11237667; doi:10.1128/msystems.01379-23)
Supplement: Supplemental Figures — Figures S1-S8. [file msystems.01379-23-s0001.pdf]

Integrative analyses on the ciliates *Colpoda* illuminate the life history evolution of soil microorganisms

Haichao Li[1,2], Kun Wu[1], Yuan Feng[1], Chao Gao[1], Yaohai Wang[1], Yuanyuan Zhang[1], Jiao Pan[1], Xiaopeng Shen[3], Rebecca A. Zufall[4], Yu Zhang[5], Weipeng Zhang[1], Jin Sun[1], Zhiqiang Ye[6], Weiyi Li[7], Michael Lynch[8], Hongan Long[*,1,2]

1. Key Laboratory of Evolution and Marine Biodiversity (Ministry of Education), Institute of Evolution and Marine Biodiversity, KLMME, Ocean University of China, Qingdao, Shandong Province, China 266003.
2. Laboratory for Marine Biology and Biotechnology, Laoshan Laboratory, Qingdao, Shandong Province, China 266237.
3. College of Life Sciences, Anhui Normal University, Wuhu, Anhui Province, China 241000.
4. Department of Biology and Biochemistry, University of Houston, Houston Texas, USA 77204-5001.
5. School of Mathematics Science, Ocean University of China, Qingdao, Shandong Province, China 266000
6. School of Life Sciences, Central China Normal University, Wuhan, Hubei Province, China 430079
7. Department of Genetics, Stanford University School of Medicine, Stanford, CA, USA 94305
8. Biodesign Center for Mechanisms of Evolution, Arizona State University, Tempe Arizona, USA 85287.

* Corresponding author, Email: longhongan@gmail.com

FIG S1
FIG S2
FIG S3
FIG S4
FIG S5
FIG S6
FIG S7
FIG S8

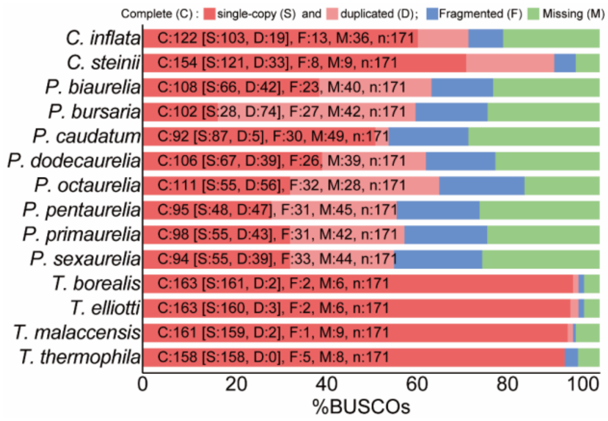

FIG S1 Quality evaluation of 14 ciliates assemblies based on BUSCO. We used alveolata_odb10 BUSCO dataset which includes 171 single copy orthologs to assess the genome assembles in this study (*C. steinii* RZ4A and *C. inflata* RL4B) and previously published ones (Table S9).

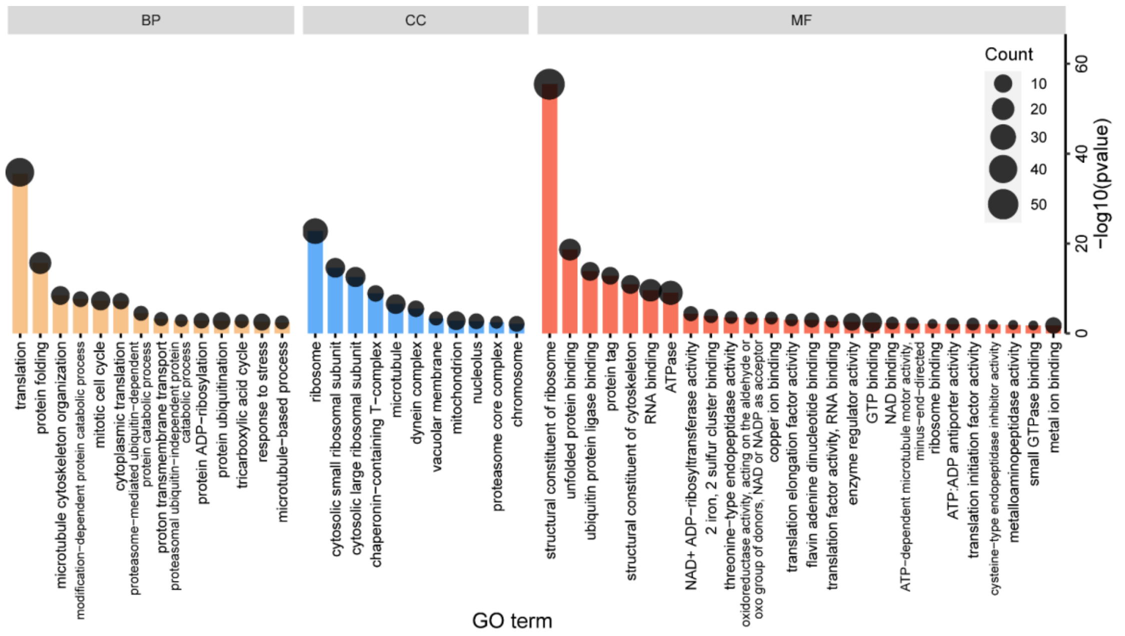

FIG S2 GO enrichment of genes from proteome sequencing on resting cysts of *C. steinii* RZ4A. BP, CC and MF represent Biological Process, Cellular Component and Molecular Function, respectively.

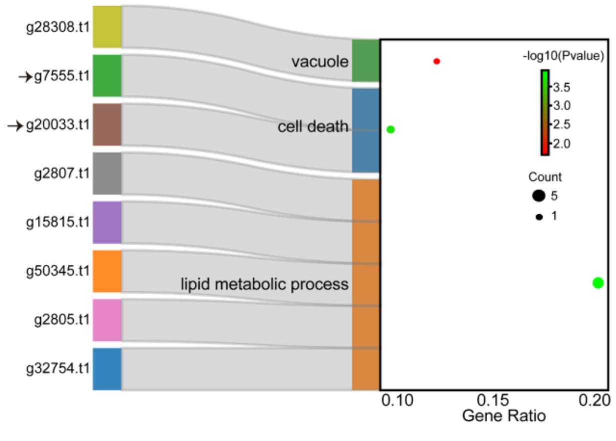

FIG S3 GO enrichment of the most significantly up-regulated genes (P value < 0.01) during the resting cysts (vs. trophonts) stage of *C. steinii* RZ4A. Gene IDs are on the leftmost of the figure. Gene Ratio represents the proportion of total DEGs out of the annotated genes in the given GO term.

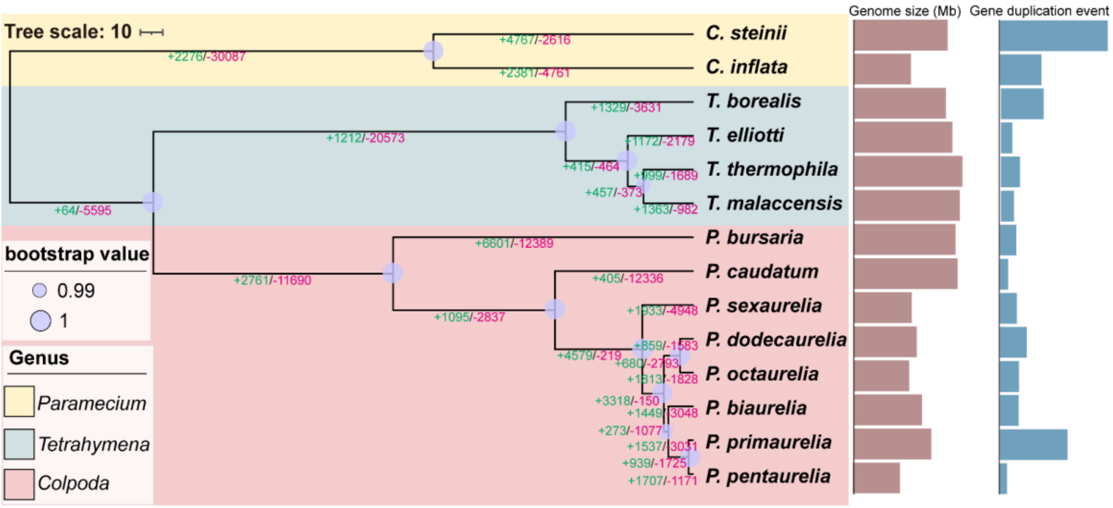

FIG S4 Phylogenomic tree showing gene family expansions and contractions. The numbers to the right of the node represent gene families of expansion (green numbers after +) and contraction (violet numbers after -). The rightmost histograms show number of gene duplication events and genome sizes.

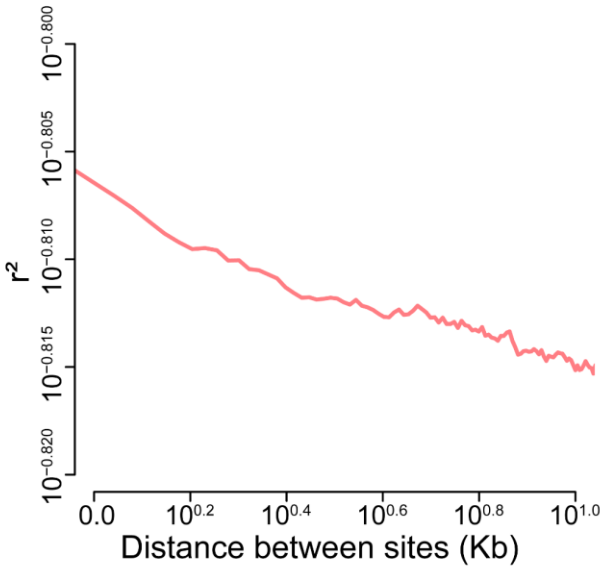

FIG S5 The decay of Linkage Disequilibrium (LD) by distance in *C. steinii*.

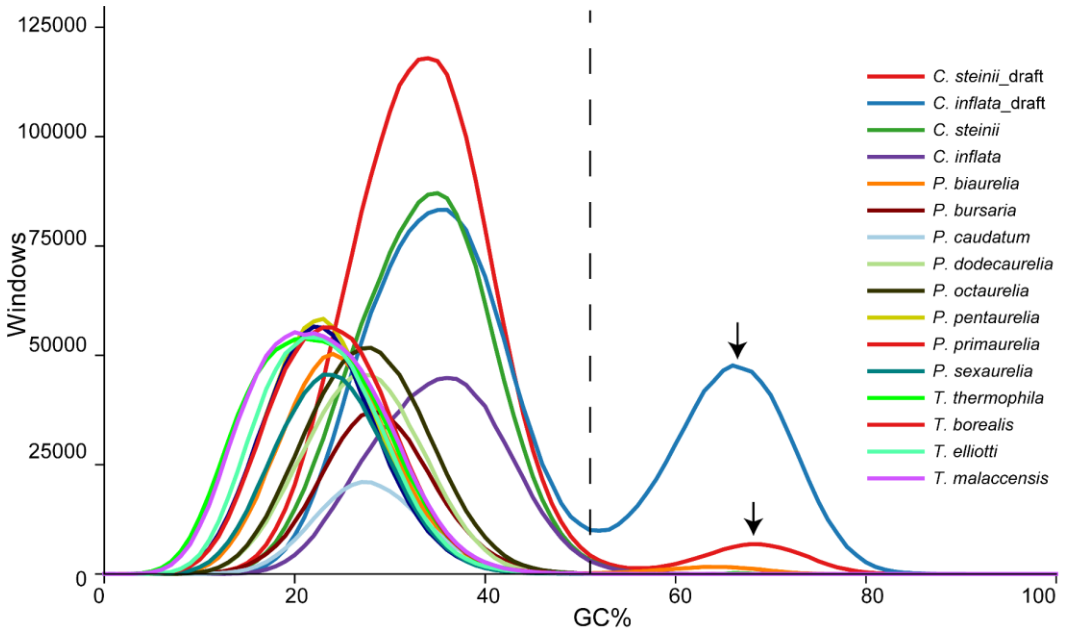

FIG S6 The GC content distributions in different ciliate genomes. Each scaffold was divided into 100bp-windows, and the y axis shows the number of windows. The arrow marked peaks are considered to be the contamination from bacterial sources. The dotted line represents 45% GC content which is the cut-off value used in the scaffold filtering.

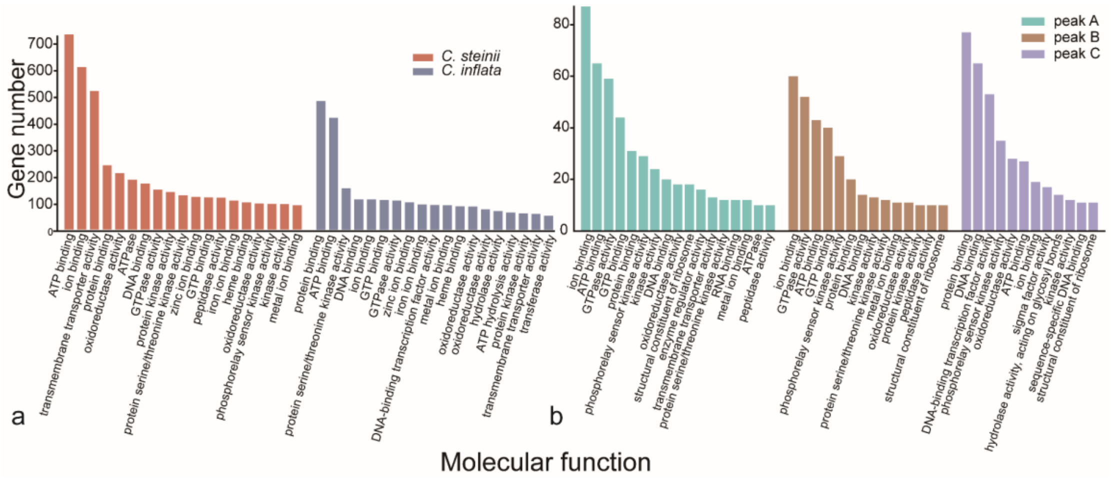

FIG S7 The molecular function of expanding and preserved genes during genome evolution. (a) Molecular function and gene number of expanded gene families in *C. steinii* RZ4A and *C. inflata* RL4B. (b) The molecular function of preserved genes in WGD events. Peaks A, B and

C are consistent with those in Fig. 4a. Peaks A and B correspond to the two WGD events in *C. steinii* RZ4A. Peak C refers to the WGD event in *C. inflata* RL4B.

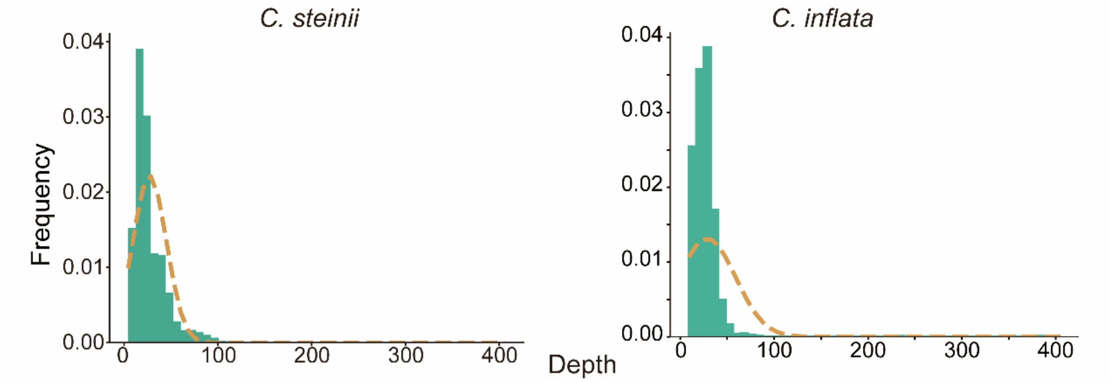

FIG S8 Histograms of sequencing depth in scaffolds of *C. steinii* RZ4A and *C. inflata* RL4B.
