# Supplementary material for: Integrative analyses on the ciliates Colpoda illuminate the life history evolution of soil microorganisms
Source: mSystems. 2024 May 31;9(6):e01379-23. doi: 10.1128/msystems.01379-23 (PMC11237667; doi:10.1128/msystems.01379-23)
Supplement: Supplemental Text — Supplemental Materials and Methods. [file msystems.01379-23-s0002.docx]

**Integrative analyses on the ciliates** ***Colpoda* illuminate the life history evolution of soil microorganisms**

Haichao Li^1,2^, Kun Wu^1^, Yuan Feng^1^, Chao Gao^1^, Yaohai Wang^1^, Yuanyuan Zhang^1^, Jiao Pan^1^, Xiaopeng Shen^3^, Rebecca A. Zufall^4^, Yu Zhang^5^, Weipeng Zhang^1^, Jin Sun^1^, Zhiqiang Ye^6^, Weiyi Li^7^, Michael Lynch^8^, Hongan Long^*,1,2^

1. Key Laboratory of Evolution and Marine Biodiversity (Ministry of Education), Institute of Evolution and Marine Biodiversity, KLMME, Ocean University of China, Qingdao, Shandong Province, China 266003.
2. Laboratory for Marine Biology and Biotechnology, Laoshan Laboratory, Qingdao, Shandong Province, China 266237.
3. College of Life Sciences, Anhui Normal University, Wuhu, Anhui Province, China 241000.
4. Department of Biology and Biochemistry, University of Houston, Houston Texas, USA 77204-5001.
5. School of Mathematics Science, Ocean University of China, Qingdao, Shandong Province, China 266000.
6. School of Life Sciences, Central China Normal University, Wuhan, Hubei Province, China 430079.
7. Department of Genetics, Stanford University School of Medicine, Stanford, CA, USA 94305.
8. Biodesign Center for Mechanisms of Evolution, Arizona State University, Tempe Arizona, USA 85287.

* Corresponding author, Email: [longhongan@](mailto:longhongan@ouc.edu.cn)gmail.com

**MATERIALS AND METHODS**

**Strain isolation and identification**

We first collected moist soil ~15 cm underground at the Yushan campus of Ocean University of China (36°3′42″N, 120°19′56″E), Qingdao, China, on August 22, 2018 (Fig. 1a to c). Then, ~5 g soil was wrapped in gauze and soaked in distilled water for one day. *Colpoda*-like single cells were picked on an Olympus SZX2-ILLTQ (Olympus, Tokyo, Japan) or a Vihent VHL745 (Vihent, Shanghai, China) dissection microscope. Single cells were cultured in 48-well cell culture plates. Species identification was done by 18S rRNA gene sequencing (PCR primers:5'-AACCTGGTTGATCCTGCCAGT-3' and 5'-TGATCCTTCTGCAGGTTCACCTAC-3' (1); PCR conditions: step1 98 °C for 30 s, step2 18 cycles of 98 °C for 10 s, 69 °C for 30 s and 72 °C for 60 s, step3 18 cycles of 98 °C for 10 s, 51 °C for 30 s and 72 °C for 60 s, step4 72 °C for 5 min) and morphological observation on a Nikon ECLIPSE Ni-U (Nikon, Tokyo, Japan) microscope. In order to purify the *C. steinii* RZ4A and *C. inflata* RL4B reference strains for *de novo* macronuclear genome assembling, we also performed five rounds of single-cell transfers (Fig. 1b). For population-level analyses, in order to preserve the natural heterozygosity, *C. steinii* strains collected across China were only single-cell transferred twice to purify, and identified by 18S rRNA (sequence identity ≥ 99.60% with the reference strains).

**Strain cultivation, DNA extraction, library preparation and sequencing**

Initially, we inoculated ~ 400 cells of *C. steinii* or *C. inflata* into 400mL of 10mM Tris-HCl medium (pH 7.5) with *Escherichia coli* MG1655 (OD600 = 0.2) as the sole food source in a 1L flask at 25 °C, 80 rpm. After 7–10 days of culture, we collected these cells when the flask contents turned clear due to the resting cyst formation, along with the exhaustion of food bacteria. Genomic DNA was extracted using the MasterPure Complete DNA&RNA Purification kit (Lucigen, Cat. No. MC85200). The extracted DNA was further purified by the Genomic DNA Clean & Concentrator^TM^-10 kit (ZYMO, Cat. No. ZRC000496). Illumina libraries were constructed by the NovaSeq^TM^ 6000 v1.5 Reagent kit (Cat. No. 20028312). Finally, 32.12 and 35.18 Gbp (PE150 mode) sequences were generated for *C. steinii* RZ4A and *C. inflata* RL4B, respectively. For the natural strains in the population genomic analyses, the extracted DNA was used to construct Illumina libraries using the TruePrep^®^ DNA Library Prep Kit V2 for Illumina (Vazyme, Cat. No. TD501), and whole genome sequencing was performed using NovaSeq 6000 at Novogene Inc. (Tianjin, China) following Li et al. (2019).

For low-input RNAseq of each life stage (trophonts in vegetative growth, reproductive cysts, resting cysts, revived trophonts), initially, 100 trophonts were inoculated into a 15 × 2.5 (diameter × depth) cm Petri dish with 100mL of 10mM Tris-HCl (pH 7.5) with *E. coli* (OD = 0.2) at 25 °C, and 3–4 replicates for each life stage of each strain. In the course of the experiment, *C. steinii* RZ4A and *C. inflata* RL4B were mostly in trophonts at 0–60h. They gradually transformed into reproductive cysts at 60–80h. After 120 hours, almost all of them became resting cysts. We then re-introduced food bacteria to the resting cysts. 12 hours later, the resting cysts began to gradually transform into trophonts. For each life stage, ~20 cells were collected and lysed, and mRNA was reverse transcribed into cDNA. cDNA 2^nd^-strand synthesis, fragmentation, amplification and library preparation were conducted following the NEBNext^®^ Single Cell/Low Input RNA Library Prep Kit for Illumina^®^ (NEB, Cat. No. E6420S) protocol. All Illumina sequencing was done by Berry Genomics, Inc. (Beijing, China).

**Oxford Nanopore Technologies (ONT) library preparation and MinION sequencing**

We first prepared 2 μg high-quality genomic DNA for each strain, using the MasterPure Complete DNA&RNA Purification kit (Lucigen, Cat. No. MC85200). Nanopore library construction was then done with the NEBNext Ultra II End-Repair/dA-tailing Module kit (NEB, Cat. No. E7546) and ligation sequencing kit (SQK-LSK109; flow-cell R9.4), and sequenced with a MinION sequencer in the lab. 36.34 and 37.93 Gbp raw bases were generated for *C. steinii* RZ4A and *C. inflata* RL4B using MinKNOW version 3.1.8, respectively.

***de novo* assembly, polishing and filtering of the macronuclear genome**

Adaptors and low-quality bases of raw Illumina reads were trimmed using fastp ver. 0.20.1 with the default settings (3). ONT sequencing reads were trimmed using Nanofilt ver. 2.8.0 with the settings “-q 9 -l 1000” (4). To filter out food bacteria contaminations (*Escherichia. coli* K-12 MG1655) and obtain clean reads, the Illumina short reads and ONT long reads were aligned against the reference genome of *E. coli* MG1655 (NCBI genome accession number: NC_000913.3) using BWA ver. 0.7.17 and Minimap2 ver. 2.17 with the default settings respectively. Then clean reads were extracted using SAMtools ver. 0.1.9 with the parameters “-bf 4” (5-7). Finally, we obtained a total of 23 Gbp nanopore and 16 Gbp Illumina PE150 sequences for *C. steinii* RZ4A; 19 Gbp nanopore and 16 Gbp Illumina PE150 sequences for *C. inflata* RL4B (Table 1). The high-quality Illumina reads were used to assess genome size and heterozygosity. Firstly, we used Jellyfish ver. 2.3 to calculate the k-mer frequency (k-mer values were 21, 33, 55, 77, 99, and 127) (8). The software GenomeScope ver. 2.0 was then used to evaluate genome size and heterozygosity based on the polyploid-aware mixture model (9). Both of the final estimated genome sizes of *C. steinii* RZ4A and *C. inflata* RL4B were 200 Mbp (Table S15). Based on the estimation, we assembled the genome by SPAdes ver. 3.13.0, and finally obtained 221 Mbp and 223 Mbp draft genomes for *C. steinii* RZ4A and *C. inflata* RL4B, respectively (Table S16) (10).

To further improve the quality of the assemblies, we performed two rounds of polishing with Racon ver. 1.4.3 and Pilon ver. 1.23 (11, 12). To filter out the contamination sequences, we downloaded all the available bacterial genome sequences from NCBI (<https://www.ncbi.nlm.nih.gov/>; January 3, 2021). Scaffolds of the polished genomes were blasted against the database using BLASTN ver. 2.10.1 (e-value 1e-5), and any scaffolds with ≥ 80% identity and accumulated hit length ≥ 60% were filtered out (13). In addition, scaffolds with GC content >45% were removed because of the distinguishing GC content distribution in the genome of bacteria and ciliates (~ 60% in bacteria and ~30% in ciliates; Fig. S6) (14). We further filtered out the micronuclear sequences as follows: suppose that DNA content is correlated with nuclear volume, the micronucleus was spherical and the macronucleus ellipsoid, after measuring their dimensions on the microscope, we calculated the volume ratio (R) of the micronucleus to the macronucleus by

$$R=\frac{\frac{4\pi r^{3}}{3}}{\frac{4\pi abc}{3}}$$

where a, b and c represent the length, width and height of the macronucleus respectively; r represents the radius of the micronucleus (a, b, c and r were measured on a fluorescence microscope after staining, Fig. 1h, m). Based on the volume ratio and the rankings of sequencing depth of scaffolds, we then identified the cutoff sequencing depth, below which was taken as micronuclear scaffolds, i.e. 4.30-fold and 8.67-fold for *C. steinii* RZ4A and *C. inflata* RL4B, respectively (Fig. S8) and removed scaffolds with lower depth.

Among the initially-excluded scaffolds during assembly filtering, we reintegrated those with both telomeres back into the assembly, as they probably represented genuine *Colpoda* chromosomes. Additionally, we also reintegrated scaffolds containing *Colpoda* ribosomal DNA, which are relatively high in G/C content and usually outliers in genomic G/C content distribution, and prone to being mistakenly filtered out by the commonly-used G/C content filter in ciliate genome assembly filtering. The final versions of the genomes (Table S16), were evaluated using QUAST ver. 5.0.2 and BUSCO ver. 4.1.4 with the settings “-l alveolata_odb10 -augustus_species Tetrahymena” (15, 16).

**Structural and functional annotations**

To identify protein-coding genes, we applied *de novo* gene prediction and transcriptome-based methods. To thoroughly recover the transcriptional profiles, we assembled transcripts with RNAseq data from three different life stages (trophonts in vegetative growth, reproductive cysts, and resting cysts) using Trinity ver. 2.8.5 with the setting “-genome_guided_max_intron 10000” (17). Then transcripts were mapped to the reference to provide genome evidences using PASA ver. 2.4.1 with the setting “-ALIGNERS blat,gmap” (18). The genome evidence was used to train a gene prediction model using Augustus ver. 3.3.3 and then genome structures were predicted (19). For further prediction, we mapped the RNAseq reads to the assembled genome to obtain gene coding regions using HISAT2 ver. 2.2.1 and then predicted genome structures by Braker2 ver. 2.1.6 and StringTie ver. 1.3.7 (20-22). The predicted genes generated by Augustus, Braker2, and StringTie were integrated to yield robust gene sets using a lab-developed Perl script. This process required that the predicted gene positions from both Braker2 and StringTie exhibit a substantial (>90%) overlap with those from Augustus. The gene sets were used as the evidence for a second round of gene training and prediction by Augustus. Finally, we identified 37,123 and 22,668 highly supported genes in *C. steinii* RZ4A and *C. inflata* RL4B, respectively.

To get gene symbol IDs, all genes were blasted with BLASTP ver. 2.10.1 against the NCBI NR database (January 5, 2021), with the setting “-evalue 1e-5 -word_size 3 -num_alignments 20 -max_hsps 20 -show_gis” (13). Gene ontology (GO) annotation was performed based on Blast2GO of OmicsBox ver. 1.4.11 with default parameters (23).

**Gene family clustering**

Orthofinder ver. 2.5.4 was used to identify the orthologous groups shared by *C. steinii* RZ4A, *C. inflata* RL4B and other ciliates (Table S9), using pairwise alignment with settings “-M msa -S diamond” (24, 25). A total of 406,576 genes derived from *C. steinii*, *C. inflata*, *T. thermophila*, *T. borealis*, *T. elliotti*, *T. malaccensis*, *P. biaurelia*, *P. bursaria*, *P. caudatum*, *P. dodecaurelia*, *P. octaurelia*, *P. pentaurelia*, *P. primaurelia* and *P. sexaurelia* were clustered into 41,111 gene families and 102,897 genes of 3,584 gene families shared by all the species. The gene families of *C. steinii* and *C. inflata* had 34,638 and 19,753 genes in orthogroups, respectively.

**Phylogenetic and gene family expansion/contraction analysis**

Based on the orthologous groups, we constructed the phylogenetic tree using single-copy genes. Briefly, each group of single-copy genes were aligned separately using MUSCLE ver. 3.8.1551 and then these genes were concatenated, after which the low alignment-quality regions (e.g., indels, mismatches, and gaps) were trimmed using Gblocks ver. 0.91b (26, 27). We constructed the phylogenetic tree using IQ-tree ver.2.1.4 with settings “-m MFP -B 1000 –bnni” (28-30). According to Jiang et al. (2019), the divergence time of *Tetrahymena* was used as the approximate calibration time point, and the divergence time for *Colpoda* species was estimated using r8s ver. 1.81 (32). The gene family expansion/contraction was processed by CAFE ver. 5.0.0 with the setting “-fixed_lambda 1” (33).

**Whole genome duplication**

For the synteny analysis of homologous gene pairs, the protein sequences of *C. steinii* RZ4A and *C. inflata* RL4B were aligned against themselves using Diamond ver. 2.0.12.150 with the setting “E-value <1e-5”. Then, we determined the syntenic blocks, estimated *Ks* values for each block, and calculated *Ks* distributions of gene pairs in collinearity blocks using WGDI ver.0.5.163 with the setting “-block_length 5” (34).

**Gene differential expression analysis**

The RNAseq datasets of different life stages were mapped to reference genomes using HISAT2 ver. 2.2.1, and the reads count of each gene was evaluated using featureCounts ver. 1.0 with the default parameters (35). Genes with significantly different expression levels were analyzed by DESeq2 ver. 1.32.0 with |fold-change| > 2, *P*-adjusted value < 0.05 (36). Gene functional enrichments were performed by clusterProfiler ver. 4.0.2 (*P* value < 0.05) (37).

**Label-free proteome quantification**

To study the proteome of resting cysts, we prepared two replicates for *C. steinii* RZ4A. We collected ~2×10^6^ resting cysts of *C. steinii* RZ4A for each replicate and extracted proteins using the 4% sodium dodecyl sulfate (Sangon Biotech, Cat. No. SB0485-500g), 100mM Tris-HCl, pH 7.6 (SDT) lysis method, and the protein was quantified by the BCA protein assay kit (P0012, Cat. No. Beyotime) (38). The enzymatic hydrolysis of protein samples was performed by the filter-aided sample preparation (FASP) method, and the salt was removed using a C18 cartridge (Waters, Cat. No. WAT023590) (38). The peptides from each sample were separated and subject to mass spectrometry analysis by nanoElute (Bruker, Bremen, Germany) coupled to a timsTOF Pro (Bruker, Bremen, Germany) equipped with a CaptiveSpray source at Lianchuan Biotechnology, Inc. (Hangzhou, China). Finally, we got the mass spectrometry (MS) data—DDA raw files which were then searched against the *C. steinii* RZ4A protein database annotated by ourselves using MaxQuant ver. 1.6.17 (39). The search followed an enzymatic cleavage rule of Trypsin/P, with a maximum of two missed cleavage sites and a mass tolerance of 20ppm for fragment ions. Carbamidomethylation of cysteines was defined as a fixed modification, whereas protein N-terminal acetylation and methionine oxidation were defined as variable modifications for database searching. The cutoff of the global false discovery rate (FDR) for peptide and protein identification was set to 0.01. Protein abundance was calculated on the basis of the normalized spectral protein intensity (LFQ intensity). Finally, we identified 1,483 genes from the proteome.

**Quantitative real-time PCR (RT-qPCR)**

To ensure the reliability of the differential gene expression analysis, we used RT-qPCR to confirm the expression patterns of 6 randomly chosen genes, of which 3 genes were significantly up-regulated and the other 3 significantly down-regulated in the resting cysts (vs. trophonts in vegetative growth). Gene-specific primers were designed using Primer Premier5 (Table S5). All reactions were performed on the StepOnePlus™ Real-Time PCR System (Thermo Fisher Scientific, Massachusetts, America) with three technical replicates. Using the Hieff UNICON® Universal Blue qPCR SYBR Green Master Mix (YEASEN, Cat. No. 11184ES08), the total volume of each reaction was 20 µL. The amplification reactions were incubated at 95°C for 5 min, followed by 40 cycles of 95°C for 10 s, 60°C for 30 s, and then the melt curves reading increased from 60 to 95°C, each time by 0.3°C. We selected gene g4956.t1 as the normalizer, whose expression level remained unchanged in the trophonts and resting cysts of *C. steinii* RZ4A. All data were comparably analyzed by the Livak method.

**SNP calling and population genetic parameter estimation**

To minimize errors in data analysis, series of filter strategies were implemented: fastp ver. 0.20.1 was used to remove adaptors and filter out low-quality bases from raw reads. Clean reads were then mapped to the reference genome using BWA ver. 0.7.17 with the default settings for bwa mem. We removed reads with multiple hits, which were possibly caused by mis-mapping, and then converted the SAM file to a BAM file using SAMtools ver. 0.1.9. Picard and GATK ver. 4.2.6.0 were used to mark and remove duplicates and filter low-quality SNPs. Finally, a vcf file was generated (40-42).

Using the curated SNPs, we estimated the population genetic parameters such as nucleotide diversity (π), Tajima's D, neutrality index (NI), and Linkage Disequilibrium (LD) Decay. We also calculated the heterozygosity of the natural lines for population analyses. Due to the high sensitivity of π and Tajima's D to sampling errors at single SNP level, we calculated these parameters by sliding-windows (200bp) along each gene by VCFTools ver. 4.2 (43, 44). The calculation of NI followed the formula of the method in Walsh and Lynch (2018). The LD Decay of *C. steinii* and heterozygosity of each population line were calculated by plink ver.1.90b6.21 (46).

**Identification of meiosis genes**

In order to build the database of meiosis genes, we searched the keywords "meiosis" and "sexual reproduction" on the website UniProt (https://www.uniprot.org/), and also referred to the meiosis genes reported (47, 48). This yielded 51 meiosis genes in total. Among them, 11 genes are meiosis-specific genes in eukaryotes, and 40 genes are meiosis-related genes. Eventually, a total of 391 homologous genes were downloaded. All the genes of *Colpoda*, *Tetrahymena* and *Paramecia* were compared with the database respectively, and the specific parameter was blastp-evalue 1e-5.

**Data visualization**

Volcano maps and GO enrichment maps were displayed by online software SRplot ([http://www.bioinformatics.com.cn/](http://www.bioinformatics.com.cn/en)). The phylogenetic tree was visualized by iTOL (<https://itol.embl.de/>). The map showing sampling sites was plotted by ggplot2 in R. Parts of the experimental flow-chart were from Servier Medical Art (https://smart.servier.com/). All statistics were done in R ver4.1.2.

**REFERENCES**

1. Medlin L, Elwood HJ, Stickel S, Sogin ML. 1988. The characterization of enzymatically amplified eukaryotic 16S-like rRNA-coding regions. *Gene* 71:491–499.

2. Li H, Wu K, Ruan C, Pan J, Wang Y, Long H. 2019. Cost-reduction strategies in massive genomics experiments. *Mar Life Sci Tech* 1:15–21.

3. Chen S, Zhou Y, Chen Y, Gu J. 2018. fastp: an ultra-fast all-in-one FASTQ preprocessor. *Bioinformatics* 34:i884–i890.

4. De Coster W, D'Hert S, Schultz DT, Cruts M, Van Broeckhoven C. 2018. NanoPack: visualizing and processing long-read sequencing data. *Bioinformatics* 34:2666–2669.

5. Li H. 2013. Aligning sequence reads, clone sequences and assembly contigs with BWA-MEM. *arXiv preprint* arXiv:1303.3997.

6. Li H. 2018. Minimap2: pairwise alignment for nucleotide sequences. *Bioinformatics* 34:3094–3100.

7. Li H, Handsaker B, Wysoker A, Fennell T, Ruan J, Homer N, Marth G, Abecasis G, Durbin R, Genome Project Data Processing S. 2009. The Sequence Alignment/Map format and SAMtools. *Bioinformatics* 25:2078–9.

8. Marcais G, Kingsford C. 2011. A fast, lock-free approach for efficient parallel counting of occurrences of k-mers. *Bioinformatics* 27:764–70.

9. Ranallo-Benavidez TR, Jaron KS, Schatz MC. 2020. GenomeScope 2.0 and smudgeplot for reference-free profiling of polyploid genomes. *Nat Commun* 11:1432.

10. Bankevich A, Nurk S, Antipov D, Gurevich AA, Dvorkin M, Kulikov AS, Lesin VM, Nikolenko SI, Pham S, Prjibelski AD, Pyshkin AV, Sirotkin AV, Vyahhi N, Tesler G, Alekseyev MA, Pevzner PA. 2012. SPAdes: a new genome assembly algorithm and its applications to single-cell sequencing. *J Comput Biol* 19:455–77.

11. Vaser R, Sovic I, Nagarajan N, Sikic M. 2017. Fast and accurate *de novo* genome assembly from long uncorrected reads. *Genome Res* 27:737–746.

12. Walker BJ, Abeel T, Shea T, Priest M, Abouelliel A, Sakthikumar S, Cuomo CA, Zeng Q, Wortman J, Young SK, Earl AM. 2014. Pilon: an integrated tool for comprehensive microbial variant detection and genome assembly improvement. *PLoS One* 9:e112963.

13. Johnson M, Zaretskaya I, Raytselis Y, Merezhuk Y, McGinnis S, Madden TL. 2008. NCBI BLAST: a better web interface. *Nucleic Acids Res* 36:W5–9.

14. Lightfield J, Fram NR, Ely B. 2011. Across bacterial phyla, distantly-related genomes with similar genomic GC content have similar patterns of amino acid usage. *PLoS One* 6:e17677.

15. Gurevich A, Saveliev V, Vyahhi N, Tesler G. 2013. QUAST: quality assessment tool for genome assemblies. *Bioinformatics* 29:1072–5.

16. Simao FA, Waterhouse RM, Ioannidis P, Kriventseva EV, Zdobnov EM. 2015. BUSCO: assessing genome assembly and annotation completeness with single-copy orthologs. *Bioinformatics* 31:3210–2.

17. Grabherr MG, Haas BJ, Yassour M, Levin JZ, Thompson DA, Amit I, Adiconis X, Fan L, Raychowdhury R, Zeng Q, Chen Z, Mauceli E, Hacohen N, Gnirke A, Rhind N, di Palma F, Birren BW, Nusbaum C, Lindblad-Toh K, Friedman N, Regev A. 2011. Full-length transcriptome assembly from RNA-Seq data without a reference genome. *Nat Biotechnol* 29:644–52.

18. Haas BJ, Salzberg SL, Zhu W, Pertea M, Allen JE, Orvis J, White O, Buell CR, Wortman JR. 2008. Automated eukaryotic gene structure annotation using EVidenceModeler and the program to assemble spliced alignments. *Genome Biol* 9:R7.

19. Stanke M, Keller O, Gunduz I, Hayes A, Waack S, Morgenstern B. 2006. AUGUSTUS: *ab initio* prediction of alternative transcripts. *Nucleic Acids Res* 34:W435–9.

20. Kim D, Paggi JM, Park C, Bennett C, Salzberg SL. 2019. Graph-based genome alignment and genotyping with HISAT2 and HISAT-genotype. *Nat Biotechnol* 37:907–915.

21. Bruna T, Hoff KJ, Lomsadze A, Stanke M, Borodovsky M. 2021. BRAKER2: automatic eukaryotic genome annotation with GeneMark-EP+ and AUGUSTUS supported by a protein database. *NAR Genom Bioinform* 3:lqaa108.

22. Pertea M, Pertea GM, Antonescu CM, Chang TC, Mendell JT, Salzberg SL. 2015. StringTie enables improved reconstruction of a transcriptome from RNA-seq reads. *Nat Biotechnol* 33:290–5.

23. Conesa A, Gotz S, Garcia-Gomez JM, Terol J, Talon M, Robles M. 2005. Blast2GO: a universal tool for annotation, visualization and analysis in functional genomics research. *Bioinformatics* 21:3674–6.

24. Emms DM, Kelly S. 2015. OrthoFinder: solving fundamental biases in whole genome comparisons dramatically improves orthogroup inference accuracy. *Genome Biol* 16:157.

25. Buchfink B, Reuter K, Drost HG. 2021. Sensitive protein alignments at tree-of-life scale using DIAMOND. *Nat Methods* 18:366–368.

26. Edgar RC. 2004. MUSCLE: a multiple sequence alignment method with reduced time and space complexity. *BMC Bioinformatics* 5:113.

27. Talavera G, Castresana J. 2007. Improvement of phylogenies after removing divergent and ambiguously aligned blocks from protein sequence alignments. *Syst Biol* 56:564–77.

28. Nguyen LT, Schmidt HA, von Haeseler A, Minh BQ. 2015. IQ-TREE: a fast and effective stochastic algorithm for estimating maximum-likelihood phylogenies. *Mol Biol Evol* 32:268–74.

29. Kalyaanamoorthy S, Minh BQ, Wong TKF, von Haeseler A, Jermiin LS. 2017. ModelFinder: fast model selection for accurate phylogenetic estimates. *Nat Methods* 14:587–589.

30. Hoang DT, Chernomor O, von Haeseler A, Minh BQ, Vinh LS. 2018. UFBoot2: Improving the ultrafast bootstrap approximation. *Mol Biol Evol* 35:518–522.

31. Jiang C, Wei W, Yan G, Shi T, Miao W. 2019. Transcriptome analysis reveals the molecular mechanism of resting cyst formation in *Colpoda aspera*. *J Eukaryot Microbiol* 66:212–220.

32. Sanderson MJ. 2003. r8s: inferring absolute rates of molecular evolution and divergence times in the absence of a molecular clock. *Bioinformatics* 19:301–302.

33. Mendes FK, Vanderpool D, Fulton B, Hahn MW. 2020. CAFE 5 models variation in evolutionary rates among gene families. *Bioinformatics* 36:5516–5518.

34. Sun P, Jiao B, Yang Y, Shan L, Li T, Li X, Xi Z, Wang X, Liu J. 2022. WGDI: A user-friendly toolkit for evolutionary analyses of whole-genome duplications and ancestral karyotypes. *Mol Plant* 15:1841–1851.

35. Liao Y, Smyth GK, Shi W. 2014. featureCounts: an efficient general purpose program for assigning sequence reads to genomic features. *Bioinformatics* 30:923–30.

36. Love MI, Huber W, Anders S. 2014. Moderated estimation of fold change and dispersion for RNA-seq data with DESeq2. *Genome Biol* 15:550.

37. Yu G, Wang L, Han Y, He Q. 2012. clusterProfiler: an R package for comparing biological themes among gene clusters. *OMICS* 16:284–7.

38. Wisniewski JR, Zougman A, Nagaraj N, Mann M. 2009. Universal sample preparation method for proteome analysis. *Nat Methods* 6:359–62.

39. Tyanova S, Temu T, Cox J. 2016. The MaxQuant computational platform for mass spectrometry-based shotgun proteomics. *Nat Protoc* 11:2301–2319.

40. McKenna A, Hanna M, Banks E, Sivachenko A, Cibulskis K, Kernytsky A, Garimella K, Altshuler D, Gabriel S, Daly M, DePristo MA. 2010. The genome analysis toolkit: a map reduce framework for analyzing next-generation DNA sequencing data. *Genome Res* 20:1297–303.

41. Weir BS, Cockerham CC. 1984. Estimating F-statistics for the analysis of population structure. *Evolution*:1358–1370.

42. Van der Auwera GA, Carneiro MO, Hartl C, Poplin R, Del Angel G, Levy-Moonshine A, Jordan T, Shakir K, Roazen D, Thibault J, Banks E, Garimella KV, Altshuler D, Gabriel S, DePristo MA. 2013. From FastQ data to high confidence variant calls: the genome analysis toolkit best practices pipeline. *Curr Protoc Bioinformatics* 43:11–10.

43. Weir BS, Hill WG. 2002. Estimating F-statistics. *Annu Rev Genet* 36:721–50.

44. Danecek P, Auton A, Abecasis G, Albers CA, Banks E, DePristo MA, Handsaker RE, Lunter G, Marth GT, Sherry ST, McVean G, Durbin R, Genomes Project Analysis G. 2011. The variant call format and VCFtools. *Bioinformatics* 27:2156–8.

45. Walsh B, Lynch M. 2018. Evolution and selection of quantitative traits. Oxford University Press, Oxford (UK).

46. Purcell S, Neale B, Todd-Brown K, Thomas L, Ferreira MA, Bender D, Maller J, Sklar P, De Bakker PI, Daly MJ, Sham PC. 2007. PLINK: a tool set for whole-genome association and population-based linkage analyses. *Am J Hum Genet* 81:559–575.

47. Chi J, Mahe F, Loidl J, Logsdon J, Dunthorn M. 2014. Meiosis gene inventory of four ciliates reveals the prevalence of a synaptonemal complex-independent crossover pathway. *Mol Biol Evol* 31:660–72.

48. Dunthorn M, Zufall RA, Chi J, Paszkiewicz K, Moore K, Mahe F. 2017. Meiotic genes in Colpodean ciliates support secretive sexuality. *Genome Biol Evol* 9:1781–1787.
