# Supplementary material for: Integrative analyses on the ciliates Colpoda illuminate the life history evolution of soil microorganisms
Source: mSystems. 2024 May 31;9(6):e01379-23. doi: 10.1128/msystems.01379-23 (PMC11237667; doi:10.1128/msystems.01379-23)
Supplement: Legend — for Movie S1. [file msystems.01379-23-s0003.docx]

Supplemental Movie S1. This movie illustrates the division process of the reproductive cysts of *C. steinii* RZ4A.
